# Global analysis of alternative splicing regulation by insulin and wingless signaling in *Drosophila *cells

**DOI:** 10.1186/gb-2009-10-1-r11

**Published:** 2009-01-29

**Authors:** Britta Hartmann, Robert Castelo, Marco Blanchette, Stephanie Boue, Donald C Rio, Juan Valcárcel

**Affiliations:** 1 Centre de Regulació Genòmica, Parc de Recerca Biomèdica de Barcelona, Dr. Aiguader 88, Barcelona, 08003, Spain; 2 Universitat Pompeu Fabra, Parc de Recerca Biomèdica de Barcelona, Dr. Aiguader 88, Barcelona, 08003, Spain; 3 Institut Municipal D'Investigació Mèdica, Parc de Recerca Biomèdica de Barcelona, Dr. Aiguader 88, Barcelona, 08003, Spain; 4 Department of Molecular and Cell Biology, University of California, Berkeley, 94720, USA; 5 Institució Catalana de Recerca i Estudis Avançats, Parc de Recerca Biomèdica de Barcelona, Dr. Aiguader 88, Barcelona, 08003, Spain; 6 Current address: Stowers Institute for Medical Research, E. 50th Street, Kansas City, 64110, USA; 7 Current address: Centre de Medicina Regenerativa de Barcelona, Parc de Recerca Biomèdica de Barcelona, Dr. Aiguader 88, Barcelona, 08003, Spain

## Abstract

**Background:**

Despite the prevalence and biological relevance of both signaling pathways and alternative pre-mRNA splicing, our knowledge of how intracellular signaling impacts on alternative splicing regulation remains fragmentary. We report a genome-wide analysis using splicing-sensitive microarrays of changes in alternative splicing induced by activation of two distinct signaling pathways, insulin and wingless, in *Drosophila *cells in culture.

**Results:**

Alternative splicing changes induced by insulin affect more than 150 genes and more than 50 genes are regulated by wingless activation. About 40% of the genes showing changes in alternative splicing also show regulation of mRNA levels, suggesting distinct but also significantly overlapping programs of transcriptional and post-transcriptional regulation. Distinct functional sets of genes are regulated by each pathway and, remarkably, a significant overlap is observed between functional categories of genes regulated transcriptionally and at the level of alternative splicing. Functions related to carbohydrate metabolism and cellular signaling are enriched among genes regulated by insulin and wingless, respectively. Computational searches identify pathway-specific sequence motifs enriched near regulated 5' splice sites.

**Conclusions:**

Taken together, our data indicate that signaling cascades trigger pathway-specific and biologically coherent regulatory programs of alternative splicing regulation. They also reveal that alternative splicing can provide a novel molecular mechanism for crosstalk between different signaling pathways.

## Abstract 2

A genome-wide analysis of the response to insulin and wingless activation using splicing-sensitive microarrays shows distinct but overlapping programs of transcriptional and posttranscriptional regulation.

## Background

Signaling pathways present a major mechanism by which cells communicate during development and as part of the normal physiology of organisms. A relatively small number of signaling pathways have been shown to regulate a large repertoire of developmental and cellular processes ranging from axis formation in the early embryo to complex immune responses. The major signaling pathways have been shown to be remarkably conserved in their components and general biological role from insects and worms to mammals [1-3]. To exert their diverse functions, these pathways are used reiteratively, mainly by regulating different transcriptional programs depending on the cellular context. The mechanisms underlying signal-regulated transcription often involve the modification of signal-transducing molecules and downstream components, ultimately affecting the potency of transcriptional regulators. Positive- and negative-acting *cis*-regulatory sequences influencing transcription have been characterized in the promoters of target genes, with targets of the same pathway sharing similar sequence motifs (reviewed in [4-8]).

The process of alternative pre-mRNA splicing expands the information content of higher eukaryotic genomes by generating multiple mature mRNAs from a single primary transcript, often with functional consequences [9-11]. It is currently clear that alternative splicing affects more than 80% of human and over 40% of *Drosophila *genes [12-14]. An increasing number of diseases are linked to misregulation of splicing or alternative splicing, emphasizing the importance of this process in the development and homeostasis of organisms [15]. Alternative splicing can affect the 5' untranslated region (UTR), open reading frame, or 3'UTR of the transcripts. Changes in the open-reading frame usually affect the protein structure, but can also regulate mRNA and protein abundance by including exons that contain premature-stop codons, which can trigger nonsense-mediated decay [16-19]. Changes in the 3' and 5'UTRs have been associated with translational efficiency and mRNA stability and can change the accessibility of microRNAs to their target sites [20].

The splicing process is catalyzed by the complex molecular machinery of the spliceosome, composed of uridine-rich small nuclear ribonucleoprotein particles and more than 100 additional proteins [21,22]. Splicing regulatory factors, including members of the Serine and Arginine-rich (SR) and heterogeneous ribonucleoprotein particle (hnRNP) protein families, modulate splice-site choice through their direct or indirect association with RNA regulatory sequence elements (splicing enhancers and silencers) present in introns and exons and influence recognition of the splice sites by the spliceosome [10,23].

Compared with the widespread effects documented on transcriptional regulation, little is known about the global impact of signaling cascades on alternative splicing (reviewed in [24-26]). Only a handful of examples of signal-induced alternative splicing have been identified and analyzed in detail. For example, cell depolarization activates calcium/calmodulin-dependent protein kinase type IV (CaMK IV), which represses a number of exons associated with a particular RNA sequence known as CaRRE responsive element [27-29]. Phorbol ester treatment of T cells promotes skipping of variable exons in the CD45 tyrosine phosphatase and inclusion of exon v5 in CD44 transcripts [30,31]. In both cases, exonic sequences have been identified that mediate these effects. In the case of CD45 exon 4, this element binds three hnRNP proteins (L, E2 and I) and acts by blocking the transition of pre-spliceosomes to fully assembled spliceosomes [32,33]. In the case of CD44 exon v5, a composite enhancer/silencer sequence mediates the repressive effects of hnRNP A1 and the activating effects of the RNA binding protein Sam68 upon its phosphorylation by ERK under conditions of T cell activation [34-36]. These examples illustrate how activation of signaling pathways can lead to a range of effects on alternative splicing regulation through distinct molecular mechanisms, including post-translational modifications of splicing factors that change their RNA binding properties, activities or subcellular localization [25,35,37].

One outstanding question, however, is to what extent signaling pathways deploy coherent programs of post-transcriptional regulation that coordinate and specify cellular phenotypes. T cell activation, for example, leads to changes in 10-15% of alternative splicing analyzed using splicing-sensitive microarrays, with the regulated genes representing a distinct set of genes and functions from those regulated at the level of transcript abundance [38].

To address this question, this study focuses on how two very different signaling pathways, the insulin and wingless pathways, affect alternative splicing regulation using a genome-wide approach. In *Drosophila*, major signaling pathways have been intensively studied and dissected both genetically as well as molecularly using tissue culture and *in vivo *systems. The insulin pathway governs metabolic changes and has been linked to growth and life span, whereas the canonical wingless pathway is involved in a diverse range of developmental decisions. While stimulation of cells with insulin induces a widespread response mediated by a cascade of protein phosphorylation events, activation of the canonical wingless pathway triggers a more linear response focused on transcriptional changes [39-44]. Our data document that both insulin and wingless pathway activation induce multiple changes in alternative splicing, affecting genes with functions coherent with the distinct roles of these pathways *in vivo*. Bioinformatic analyses of the target genes identified two sequence motifs enriched near regulated 5' splice sites. Our results illustrate how signaling pathways can trigger a coherent set of alternative splicing events relevant for cell growth and differentiation of diverse cell types.

## Results

### Transcriptional changes induced upon activation of the insulin and wingless pathways in S2 cells

**Figure 1:**
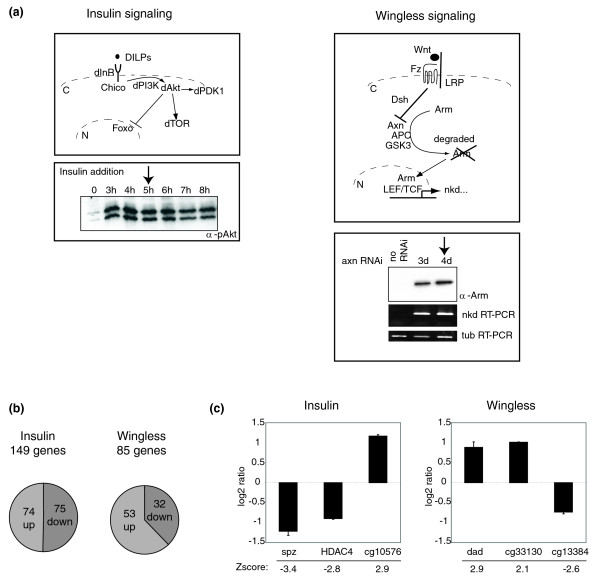
Activation of insulin and wingless signaling pathways in *Drosophila *S2 cells. **(a) **Schematic representation of the insulin and wingless signal transduction cascades and controls of their activation in our experimental system. Key protein components and their interactions for each pathway are schematized. Dashed lines represent cell and nuclear membranes. C and N indicate cytoplasm and nucleus, respectively. Stimulation of insulin signaling from 0-8 h was monitored by western blotting using an anti-phospho-Akt antibody (left panel). Activation of the wingless pathway, achieved through RNA interference (RNAi)-mediated depletion of axin (axn), resulted in the nuclear accumulation of Armadillo (Arm) as assessed by western blot analysis and activation of a known target gene, *naked cuticle *(*nkd*) monitored by RT-PCR (right lower panel). Amplification of *tubulin *(tub) transcripts served as loading control. The arrow indicates the time-point used for our microarray analysis. **(b) **Distribution of genes showing transcriptional up- and down-regulation upon activation of insulin and wingless. **(c) **Validation of microarray predictions by quantitative RT-PCR. Three genes are shown for each pathway. Results are presented as log2 ratio of signals obtained under conditions of pathway activation and controls. Z-scores predicted by microarray data analysis are indicated below the graphs.

Binding of insulin-like peptides to the insulin receptor in *Drosophila *cells leads to the activation of dPI3 kinase, which in turn activates dAkt kinase, which triggers a wide variety of responses and effects on other pathways (Figure 1a) [45,46]. *Drosophila *S2 cells were treated with 30 μg/ml human insulin and pathway activation was monitored using a phospho-epitope specific antibody against phosphorylated dAKt kinase. Phospho-dAKt was observed as soon as 20 minutes after insulin treatment (not shown) and persisted for at least 8 hours, consistent with previous studies (Figure 1a, bottom left) [47]. Guided by previous analysis of transcriptional targets [48,49], and to allow RNA turnover and minimize indirect effects after insulin activation, total RNA was isolated 5 hours after insulin treatment.

Activation of the canonical wingless pathway stabilizes Armadillo, the *Drosophila *beta-catenin homologue, preventing its degradation by a multiprotein complex containing axin. This results in the nuclear accumulation of Armadillo which, together with LEF/TCF transcription factors, regulates transcription of target genes (Figure 1a) [8,50]. Efficient activation of the wingless pathway can be achieved by reducing the levels of *axin *mRNA by RNA interference for 3-4 days [41]. Treatment of S2 cells with double-stranded RNA (dsRNA) against *axin *for 4 days resulted in a significant increase in the levels of Armadillo protein and of one of its regulated mRNA targets (*naked cuticle *(*nkd*); Figure 1a, bottom right). For our analysis, total RNA was isolated at this 4 day time point.

To monitor transcriptional and alternative splicing changes induced by activation of the insulin and wingless pathways, a custom-designed microarray platform was employed featuring probes for all *Drosophila *genes for which different mRNA isoforms generated by alternative splicing have been described [51]. Three biological replicates of total RNA isolated after pathway activation or controls (untreated cells for insulin, control dsRNA for wingless) were purified, reverse transcribed into cDNA and labeled with Cy5 or Cy3 fluorochromes; after hybridization of the cDNA to the microarray, the ratio of fluorescence between the Cy5 and Cy3 signals was measured, normalized and a Z-score (measuring the statistical confidence of the fold-change observed in the microarrays) was determined for the three biological replicates [51].

As expected, activation of either signaling pathway in S2 cells led to a significant number of transcriptional changes (Figure 1b), with 149 genes affected by activation of the insulin pathway and 85 genes affected by wingless activation. The transcriptional effects detected by the microarray were independently validated using quantitative real-time PCR for eight genes of each pathway, with validation rates of over 90%. Figure 1c shows validation of predicted transcriptional up- and down-regulation for three genes in each pathway. Log2 ratios refer to the changes in mRNA abundance determined by real-time PCR. While some of the detected changes had been reported previously (for example, *notum*, *frizzled 2*), the majority of the changes observed in our array experiments represent novel target genes of the insulin and wingless pathways (Additional data file 1).

### Numerous changes in alternative splicing patterns upon insulin and wingless activation

**Figure 2:**
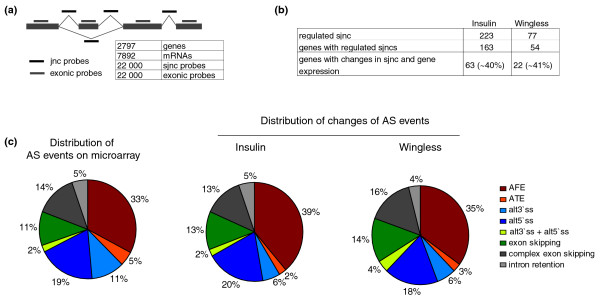
Numerous changes in alternatively spliced mRNA isoforms induced by insulin and wingless. **(a) **Features of microarray design. The array contains 36-mer probes complementary to each exon and splice junction (sjnc) for all annotated *Drosophila *genes for which there is evidence of alternative splicing. The number of genes, mRNAs and probes present in the array are indicated. **(b) **Summary of regulated junctions and genes detected upon activation of insulin and wingless pathways. **(c) **Distribution of classes of alternative splicing events for all *Drosophila *genes (left) and for those regulated by insulin (middle) and wingless signaling (right). AFE, alternative first exon; ATE, alternative terminal exon; alt3(5)'ss, alternative 3(5)'splice site.

**Figure 3:**
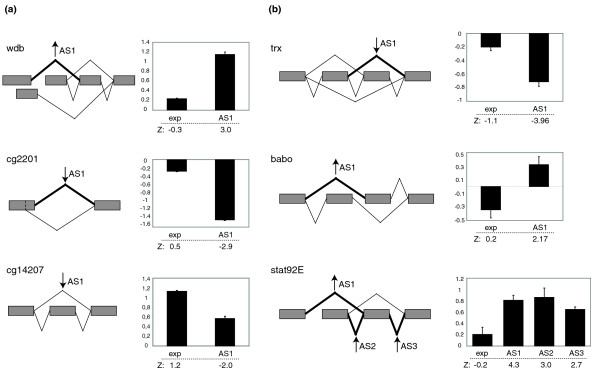
Validation of microarray-predicted changes in splice junctions using quantitative RT-PCR. Examples of alternative splicing patterns regulated by **(a) **insulin and **(b) **wingless signaling are shown. For each gene, a primer pair was designed to amplify a constitutive part of the transcript, thus monitoring general changes in transcription (exp). In addition, primer pair(s) in which one of the primers covers a splice junction were used to amplify and monitor changes in expression of particular isoforms, as indicated. Changes in splice junctions were evaluated relative to the change in gene transcription. RT-PCR results are presented as log2 ratio of eCp values obtained under conditions of pathway activation and controls. The corresponding Z-score values from the microarray prediction are indicated below the graphs for each event. Various classes of alternative splicing events are detected, including alternative first exons, alternative 5' or 3' splice sites, cassette and mutually exclusive exons and more complex patterns. In some cases, expression changes are not significant and alternative splicing changes are detected in the absence of significant changes in expression (for example, *wdb*, *cg2201*, *trx*, *stat92E*). In others, changes in splice junctions are clearly distinct from changes in expression (for example, *cg14207*) or even occur in the opposite direction (for example, *babo*). In some instances, changes in one splice junction probe monitoring a particular spliced isoform are not reciprocated by converse changes in probes monitoring the alternatively spliced product. This suggests the existence of additional processing pathways. Indeed, semi-quantitative RT-PCR using primers external to some of the alternatively spliced regions frequently detects the existence of additional, non-annotated isoforms (data not shown).

To monitor changes in alternative splicing, the microarrays contain probes covering each reported exon-exon junction (splice-junction), both constitutive (present in all annotated isoforms) or alternative (specific of only particular isoforms), as well as exon-specific probes (Figure 2a) [51]. This design allowed us to monitor a variety of alternative splicing events, including cassette exons, alternative 5' and 3' splice sites, alternative first exon usage (indicative of alternative promoters) and alternative 3' termination sites. An important issue in splicing microarray analysis is to distinguish real splicing changes from changes in transcripts caused by a quantitative change in gene expression. We define a splicing change as a replicated change in the relative signal associated with a splice junction probe between two conditions, which is statistically distinguishable (through its Z score) from the signals from other probes in the array and from the average change of all other probes monitoring other splice junctions and exon probes (constitutive or alternative) in the same transcript (which we assume reflects overall expression levels). A significant number of changes in splice junction probes were observed upon activation of either pathway and, as observed for transcriptional changes, activation by insulin resulted in more extensive changes than activation of the wingless pathway (Figure 2b). Over 150 genes showed changes in at least one splice junction in insulin-treated cells and 54 genes showed splice junction changes upon wingless pathway activation (Additional data file 2). Interestingly, a similar fraction (around 40%) of the genes showing changes in alternative junction probes also showed changes in general expression of the gene (see below). In these cases, the fold differences between probes monitoring transcriptional changes and alternative splicing changes were, however, sufficiently significant as to document the occurrence of changes in splicing patterns. To validate the changes in alternative splicing predicted by the microarray results, quantitative RT-PCR assays were performed using two primer pairs, one monitoring expression of constitutive exons (that is, general transcript levels) and another pair measuring changes in exon-exon junctions, to monitor expression of particular isoforms (see Materials and methods). As for the microarray data, changes in alternative splicing were scored as significant differences between changes in gene expression and changes in particular isoforms. Quantitative RT-PCR assays were carried out for 15 different genes, of which 11 (70%) were validated. Figure 3 shows the results obtained for six of these genes and their associated alternative splicing events.

The microarray contains approximately the same number of constitutive and alternative splice junction probes. As expected, a larger number of alternative splice junction probes showed changes upon activation of the insulin and wingless pathways compared with constitutive junction probes. The number of constitutive probes showing changes after gene expression normalization was, however, significant (up to 30%, not included in our analysis), suggesting that some of these junction probes may monitor non-annotated alternative isoforms. Indeed, RNA analyses using semi-quantitative RT-PCR detected novel isoforms for 3 of the 15 genes analyzed (data not shown).

Figure 2b shows the distribution of alternative splicing classes among the changes observed upon activation of the insulin and wingless pathways, as well as the distribution of alternative splicing classes among the splicing events featured in the whole microarray. The overall distribution (total, insulin, wingless) is similar for intron retention events (5%, 5%, 4%), exon skipping (11%, 13%, 14%), complex exon skipping events (14%, 13%, 16%) and a combination of alternative 3'- and 5'-splice-site events (2%, 2%, 4%). Changes in alternative splicing induced by these signaling pathways seem to affect a lower proportion of alternative 3' splice sites (11%, 6%, 6%) and alternative terminal exons (5%, 2%, 3%) while certain increases in alternative first exons is observed, at least for insulin (33%, 39%, 35%) (Figure 2c). The latter could be due to changes in promoter usage as a consequence of transcriptional changes induced by activation of these pathways.

Indeed, 40% of the genes showing changes in splice junction also show changes at the transcriptional level, suggesting a link between transcription and splicing in genes regulated by these signaling pathways. Interestingly, however, the use of alternative first exons is not systematically linked to transcriptional changes: only 30% of insulin genes or 36% of wingless genes using alternative promoters also show overall changes in transcript abundance (data not shown). This suggests that qualitative changes in transcript structure and splicing patterns, rather than quantitative changes in transcript abundance, are a frequent regulatory outcome of activation of these pathways. For about 7% of the genes with changes in alternative promoter usage, changes in alternative splicing are observed that affect regions of the pre-mRNA located at a significant distance from the promoters, suggesting the possibility that promoter choice can have durable consequences on splice site choices. Taken together, these observations are consistent with the emerging concept that coupling between transcription and splicing can influence changes in alternative pre-mRNA processing [52,53] and suggest that co-transcriptional splicing can play a mechanistic role in mediating the effects of insulin and wingless on alternative splicing.

### Functional overlap of genes regulated at the levels of transcription and alternative splicing

In an attempt to address the functional relevance of the observed changes in alternative splicing, gene ontology (GO) overrepresentation analyses were carried out for the genes that show transcriptional changes and changes inalternative splicing and those showing exclusively changes in alternative splicing, using as a reference the complete set of genes covered by the microarray. GO terms were subsequently grouped in broad functional related categories and the proportion of enriched GO terms compared to the overall number of enriched terms for each pathway is represented in Tables 1 and 2. One first insight was that the two pathways showed distinguishable profiles of GO categories, both for genes experiencing transcriptional changes and for those genes showing changes in alternative splicing. These results suggest that, as is the case for transcriptional regulation, alternative splicing deploys a distinct regulatory program characteristic of each signaling pathway. A second conclusion was that some of the most populated functional categories of enriched GO terms are shared between transcriptional and post-transcriptional regulation, and these shared categories are characteristic for each pathway. In the case of wingless-regulated genes, functions related to signal transduction (including lipid - for example, phospholipid - metabolism) as well as learning, memory and olfaction-related genes were among the enriched categories, at both the transcriptional and post-transcriptional levels. Consistent with one key function of insulin signaling, genes with functions in carbohydrate, amino acid and intermediary metabolism constitute a prominent category of insulin-regulated genes, both transcriptionally and post-transcriptionally. Similar gene ontology enrichment was observed when the analysis included genes showing changes only in alternative splicing but not in transcript levels. The broad category of genes involved in developmental processes and decisions shows changes for both pathways and regulatory mechanisms, although GO terms characteristic of each pathway (for example, development of the tracheal system for insulin) could be identified. Collectively, these results strongly suggest that the changes in alternative splicing triggered by insulin and wingless are biologically meaningful and functionally coherent with the well-studied transcriptional regulation programs deployed by these signaling pathways (see Discussion).

**Table 1:** Summary of Gene Ontology overrepresentation analysis of genes regulated by insulin

GO term category	Transcription (69)	AS (40)	AS only (36)
Carbohydrate, amino acid and intermediate metabolism	18 (26%)	14 (35%)	11 (30%)
Immune response (including antifungal, antibacterial)	6 (9%)	2 (5%)	0
Developmental decisions (including tracheal system)	23 (33%)	5 (12%)	0
Microtubule organization	0	2 (5%)	8 (22%)
Cell death	3 (4%)	0	0
Behavior, olfaction, memory, learning	0	3 (7%)	0
RNA metabolism	0	3 (7%)	1 (3%)
Signal transduction, lipid metabolism	1 (1%)	0	0

GO overrepresentation analysis of the function (biological process) of genes regulated transcriptionally and at the level of alternative splicing by insulin. GO terms were grouped in broad functional categories and the number of enriched GO terms in each category is indicated. Also indicated is the percentage that each number of enriched GO terms represents from the total number of enriched terms (indicated at the top) for the classes of genes showing transcriptional changes, alternative splicing (AS) changes or alternative splicing without transcriptional changes (AS only). Only GO term categories with a *p*-value < 0.05 are represented.

**Table 2:** Summary of Gene Ontology overrepresentation analysis of genes regulated by the wingless pathway

GO term category	Transcription (45)	AS (32)	AS only (38)
Signal transduction, lipid metabolism (for example, phospholipid metabolism)	14 (31%)	8 (25%)	5 (13%)
Learning, memory, behavior, olfaction	5 (11%)	7 (22%)	3 (8%)
Developmental decisions	10 (22%)	5 (16%)	11 (29%)
Cell death	3 (7%)	2 (6%)	2 (5%)
Carbohydrate, amino acid and intermediate metabolism	0	1 (3%)	2 (5%)
Immune response	0	0	0
RNA metabolism	0	0	0
Microtubule organization	0	0	0

GO overrepresentation analysis of the function (biological process) of genes regulated transcriptionally and at the level of alternative splicing by activation of the wingless pathway. GO terms were grouped in broad functional categories and the number of enriched GO terms in each category is indicated. Also indicated is the percentage that each number of enriched GO terms represents from the total number of enriched terms (indicated at the top) for the classes of genes showing transcriptional changes, alternative splicing (AS) changes or alternative splicing without transcriptional changes (AS only). Only GO term categories with a *p*-value < 0.05 are represented.

### Signal-regulated alternative splicing as another level of pathway regulation and crosstalk between pathways

Signaling by the wingless pathway plays a role in diverse developmental processes and frequently involves autoregulation and extensive crosstalk with other signaling pathways. For example, patterning of the wing imaginal disc is achieved mainly through the interplay of the transforming growth factor (TGF)β, Wingless, Notch and Hedgehog signaling pathways [39,54-56]. Therefore, we considered the possibility that modulation of pathway activity through autoregulation, or crosstalk between pathways could also be affected by changes in alternative splicing of the genes involved. Indeed, signaling genes were among the enriched categories of differentially spliced genes upon activation of the wingless pathway (Table 1) and changes in alternative splicing of several key genes involved in wingless signaling, including the wingless receptor *frizzled2 *(*fz2*) [57] and the wingless modifier *rotund *(*rn*), were found [58] (Tables 3 and 4). Equally interesting, changes in alternative splicing of genes important for TGFβ and JAK-STAT signaling pathways were also detected (Tables 3 and 4), including an alternative splicing event in the activin receptor *baboon*, which is predicted to affect ligand binding, and another functionally important event in the Signal transducer and activator of transcription protein 92E (*stat92E*) [59], which affects dimerization of the protein on its target DNA (Figure 3b). Taken together, these results show that activation of the wingless pathway results in alternative splicing changes that can mediate or modulate the wingless pathway itself or the crosstalk between pathways.

**Table 3:** Examples of genes encoding signaling pathway components that show changes in splice junctions upon wingless pathway activation

Gene name	Type of AS	Effect of AS	Expression	Function
*Fz2*	Alternative promoter	Alternative 5' UTR	Upregulated	Wnt receptor activity
*dawdle*	Alternative promoter	Alternative 5' UTR	Upregulated	TGF-β receptor binding
*baboon*	Mutually exclusive exons	Alternative activin receptor domain	No change	TGF-β type I receptor
*stat92E*	Alternative promoter; exon skipping	Alternative stat interaction domain	No change	JAK/STAT signaling
*hr51*	Multiple exon skipping	Alternative coding sequence	No change	Steroid hormone receptor

Pathway components are described, together with the type of alternative splicing event, predicted consequences for the transcript/protein and function in the pathway (as retrieved from Flybase and literature). AS, alternative splicing.

**Table 4:** Examples of genes encoding modulators of signaling pathways that show changes in splice junctions upon wingless pathway activation

Gene name	Type of AS	Effect of AS	Expression	Function
*rotound*	Exon skipping	Alternative coding sequence	No change	wingless expression regulation
*syndecan*	Alternative 3' splice site	Alternative 5' UTR	Upregulated	Heparan sulfate proteoglycan
*IP3k2*	Alternative 5' splice site	Alternative 5' UTR	Upregulated	Inositol 3P 3-ki-nase activity
*sprint*	Alternative promoter; Alternative polyadenyl.	Alternative VPS9 and Ras-association	Potentially upregulated	Ras GTPase binding
*cdep*	Exon skipping	Alternative Ferm_3 domain	No change	Regulation of Rho signaling
*pink1*	Alternative 3' splice site	Alternative 5' UTR	No change	Serine/threonine kinase
*CG15611*	Exon skipping	Alternative coding sequence	Downregulated	Regulation of Rho signaling
*smi35A*	Complex exon skipping	Alternative 5' UTR	Upregulated	Tyr-phosphorylation regulated kinase
*eip63E*	Alternative promoter	Alternative 5' UTR and coding sequence	Upregulated	Cyclin-dependent protein kinase

Modulators of signaling activities are described, together with the type of alternative splicing event, predicted consequences for the transcript/protein and function in the pathway (as retrieved from Flybase and literature). AS, alternative splicing.

### Pathway-specific enrichment of sequence motifs in the vicinity of regulated junctions

**Figure 4:**
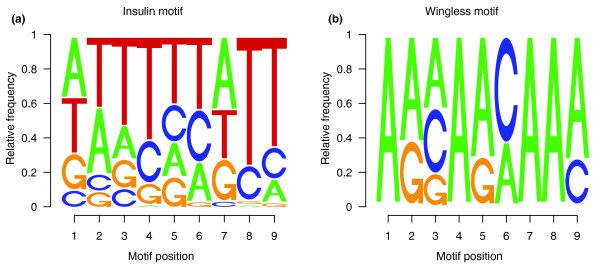
Overrepresented sequence motifs present at the 5' end of intronic regions associated with splice junctions regulated by the **(a)** wingless and **(b)** insulin pathways. Motifs were derived from a dataset of sequences corresponding to the 50 nucleotides of introns flanking splice junctions that change upon activation of a signaling pathway, as well as the corresponding regions in the same intron of the other 11 *Drosophila *species. Motifs were identified using MEME and PHYLOGIBBS software and the specificity of the enrichment assessed with a set of control sequences derived from constitutive and alternative splice junctions that do not change upon activation of the signaling pathway. A detailed account of motifs and statistical assessment of their significance can be found in [63]. Represented are the relative frequencies of each nucleotide at each position in the nine nucleotide motifs. Genes containing the junctions included in each of the motifs are as follow. Insulin motif (44): *sbb*, *cg15611*, *graf*, *cg7995*, *cg13213*, *cul-2*, *cher*, *ald*, *cg6265*, *cg7950*, *cg1021*, *cg7059*, *tomosyn*, *cg8036*, *cg1141*, *wdb*, *cg3168*, *cg8789*, *cg32425*, *cg16833*, *cg13499*, *cg4502*, *cg31732*, *cg32103*, *cg33085*, *sesB*, *scb*, *sdc*, *nemy*, *Ef2b*, *keap1*, *drpr*, *cg15105*, *: cg5059*, *spi*, *cg6231*, *cg14869*, *cpx*, *spri*, *cg16758*, *dom*, *Ca-P60A*, *ptp99A*, *cg33130*. Wingless motif (10): *stat92E*, *trx*, *cg2747*, *smi35A*, *hph*, *ced-6*, *cg33130*, *slo*, *cg4502*, *cg5794*.

A computational search for sequence motifs enriched near splice junctions regulated by the insulin and wingless pathways was carried out. For each of the two sets of differentially regulated junctions, intronic regions of 50 nucleotides flanking each junction were selected, together with the orthologous regions in the other 11 *Drosophila *species [60]. Motif searches within each set of sequences were carried out using MEME [61] and PHYLOGIBBS [62] software, aiming at identifying motifs enriched in each set of sequences for which there is evidence of phylogenetic conservation. This generated a panel of putative motifs [63]. Two significant motifs were identified, a uridine-rich motif associated with junctions regulated by insulin (identified through PHYLOGIBBS; Figure 4a) and an adenosine-rich motif associated with junctions regulated by the wingless pathway (identified through MEME; Figure 4b). These motifs were significantly enriched compared with their distribution in sets of control regions of comparable size derived from either constitutive or non-constitutive junctions that did not show differential regulation by the wingless or insulin pathways [63]. We propose that these motifs are part of the *cis*-acting elements through which signaling pathways regulate alternative pre-mRNA splicing.

## Discussion

High-throughput methods for gene expression analysis are providing unprecedented opportunities to study cellular programs of transcriptional and post-transcriptional regulation. Although the detection of splicing variants requires an additional level of sophistication in data analysis, important new insights into splicing regulation have been gathered through the use of large scale sequence alignments, microarrays and proteomics (reviewed in [9,10,64]). One common theme emerging from these pioneering studies is that changes in alternative splicing and changes in transcription affect largely independent sets of genes [38,65,66]. For example, analysis of pairs of major mouse tissues using genome-wide splicing-sensitive microarrays concluded that only 15-20% of genes regulated at the level of splicing were also regulated at the level of transcript abundance, an overlap that may not differ from statistical random sampling [65]. The same conclusion was reached in a comparative analysis of human and chimpanzee tissues [64]. Similarly, the majority of genes showing changes in alternative splicing in Jurkat cells activated by phorbol esters do not show changes in transcript levels [38]. The implication of these results is that programs of gene regulation that induce transcriptional changes and those that modulate the levels of splice variants are established independently, perhaps to coordinate different aspects of cell differentiation, response to environmental stimuli, and so on. In contrast, a study using a splicing array design dedicated to 1,500 genes relevant for prostate cancer showed that 60-70% of genes experiencing changes in splicing in prostate tumor biopsies also showed changes in transcript levels [67,68]. These different figures may arise from differences in experimental setup or in the sensitivity of the analytical methods utilized, but they may also reflect different extents of coupling between transcription and RNA processing in different biological situations (for example, coupling may be more prominent in prostate gene regulation or in disease samples than in terminally differentiated tissues).

Our results suggest an intermediate situation for the response to signaling pathways in *Drosophila *cells. We find that 40% of genes changing in alternative splicing also show changes in transcript levels upon activation of the insulin pathway or the wingless cascade (Figure 2b). Given the significant transcriptional effects of activation of these pathways, it is perhaps not surprising that coupling between transcription and splicing will be prominent in signaling responses. Coupling can reflect effects on alternative splicing brought about by quantitative changes in transcriptional level of a gene. In these cases, changes in alternative splicing may be caused by titration of limiting splicing factors, differences in splicing factors recruited co-transcriptionally, or changes in transcription elongation rates. Solid precedents exist for such forms of transcriptional/post-transcriptional coupling (reviewed in [53]). In addition, changes in spliced isoforms can be linked to selection of alternative promoters and transcription start sites. Our results suggest that coupling of transcription and alternative splicing upon activation of signaling pathways in *Drosophila *employ both changes in transcript structure (alternative promoter usage, which apparently can have long-range effects on downstream events) as well as changes in transcript levels. The latter show also similar average fold changes in transcript levels and in splice site selection. These changes are relatively modest (around twofold) but consistent across experimental setups, timings and pathways [38]. Coordinated changes in transcription and alternative splicing may be important to quickly deploy changes in gene expression that will help the cell to adapt to new functions induced by insulin or wingless stimulation. Another mechanism by which alternative splicing can influence transcript levels is the generation of premature stop codon-containing transcripts through alternative splicing, which leads to RNA degradation through the Nonsense Mediated Decay (NMD) pathway [69]. This could affect 7-9% of alternative splicing changes in our dataset, although evidence against widespread coupling between alternative splicing and NMD has been reported in mammalian cells [65].

A key question is the extent to which these changes in alternative splicing are biologically meaningful, an issue relevant for alternatively spliced transcripts in general [9-11]. Previous genome-wide studies stress the largely independent functions of genes regulated at the transcriptional and post-transcriptional levels [38,64,65]. The implication of these results is that different layers of gene regulation deploy different programs of functional activities. For example, in response to phorbol ester-mediated T cell activation, transcriptional changes target genes associated with immune response and cytoskeletal architecture, while alternative splicing changes are often associated with regulation of the cell cycle [38].

Our results on both signaling pathways indicate that some categories of enriched GO terms are distinct for transcription and splicing regulation, consistent with these previous observations. The majority of the most populated categories of enriched GO terms, however, show a substantial coincidence between transcription and/or alternative splicing (Tables 1 and 2). This convergence of gene functions is a common feature of both pathways analyzed, despite the fact that the categories of genes regulated by each pathway are significantly different. Insulin targets various genes involved in carbohydrate, amino acid and intermediary metabolism, consistent with known functions of this hormone in cellular homeostasis. Wingless targets genes relevant for long-term potentiation, memory formation and olfaction, which is intriguing given the non-neural phenotype of S2 cells. Additional functions include components and regulators of signaling pathways as well as membrane lipid metabolism (for example, phospholipid metabolism, relevant to activation of various signaling routes), which would be consistent with the morphogenetic functions of the pathway and also suggests a novel layer of mechanisms for crosstalk between pathways (see below).

Why should insulin and wingless signaling put together a coherent transcriptional and post-transcriptional program of gene regulation targeting similar classes of genes, while terminally differentiated tissues and phorbol ester-induced T cells deploy distinct regulatory programs affecting different classes of genes? One obvious contributor to this difference is the larger overlap/coupling between transcription and splicing in insulin and wingless signaling discussed above. Coherent gene functions, however, are also generally observed between the subsets of genes that show changes in just alternative splicing (fourth columns in Tables 1 and 2). Furthermore, substantial overlap in functions remains upon removal of genes showing changes in alternative promoter usage (about 20% of the genes for either pathway) from the GO analyses (Additional data file 3). Fast responses to insulin and wingless stimulation may require a focused response that exploits the repertoire of gene regulation mechanisms available to the cell to build up a change in cell phenotype or homeostasis. While differences in the experimental protocols utilized to activate each pathway could influence the outcome of our experiments, the similarity of the overall conclusions obtained for the two pathways, which differ both in biological function and in the range of their molecular effects, suggests that deploying coherent functions in transcriptional and post-transcriptional programs may be a general feature of signaling cascades. In any case, our observations argue that full understanding of the response to these and other signaling pathways will require exploring both transcriptional and post-transcriptional regulation.

Another relevant case can be made for the alternative splicing changes induced by wingless activation on components of its own pathway as well as other pathways, suggesting feedback control and crosstalk between signaling routes. It is well established that signaling pathways interact extensively to achieve growth, differentiation and developmental patterning events in which wingless plays a pivotal role. For example, in the wing imaginal disc, Wingless, Hedgehog and Decapentaplegic act as morphogens specifying cell-fates along the axes [39,54,55,70,71]. It was shown that an enhancer-region in the gene *vestigial *(*vg*), a selector gene that defines the wing primordium, combines inputs from short-range Notch signaling across the dorso-ventral compartment boundary and signals from the long-range morphogens Wingless and Decapentaplegic ([56] and references therein). Another prominent example is the eye imaginal disc, the precursor of the eye. Temporal coordination of inputs from the Hedgehog, Wingless, Decapentaplegic, Notch, Receptor Tyrosine Kinase (RTK) and JAK-STAT signaling pathways pattern the eye (reviewed in [72]). Using *Drosophila *genetics, it was shown that the JAK/STAT pathway promotes the formation of the eye field through repression of the *wingless *gene and that this depends on Stat92E [73]. Our observation that wingless activation causes changes in alternative splicing of *stat92E *suggests the interesting possibility that the two pathways influence each other through transcriptional and post-transcriptional effects. It will be of great interest to investigate the underlying molecular mechanisms and, more generally, address the impact of alternative splicing regulation in these pathways.

What could be the molecular mechanisms that mediate the changes in alternative splicing triggered by insulin and wingless? Changes in the levels of general factors are known to modulate splice site choice [10,23]. No clear changes in expression or alternative splicing of the 90 RNA binding proteins featured in the array were observed upon activation of either pathway. It is possible, however, that changes in subcellular localization of splicing factors alter their functional levels in the nucleus [37]. These or other changes in activity of splicing regulators are likely to be brought about by post-translational modifications induced by signaling cascades. Indeed, previous data suggest that both signaling cascades possess the ability to regulate alternative splicing changes through interactions or modifications of splicing factors. For example, insulin in vertebrates has been shown to regulate alternative splicing of protein kinase C (PKC)-beta through phosphorylation of the SR protein Srp40 [74,75]. There is also evidence for a role of beta-catenin, a wingless pathway effector, in alternative splicing regulation: changes in beta-catenin levels lead to alternative splicing changes, which may be mediated by splicing factors that have been reported to interact with beta-catenin [76,77].

Similar mechanisms, involving modifications of splicing regulators and the *cis*-acting sequences from which they act (Figure 4), are likely to mediate the changes in alternative splicing reported here. Identification of widespread splicing changes affecting genes with functions coherent with the distinct roles of these pathways *in vivo *is an important first step to unravel these mechanisms.

## Conclusion

The results presented in this study document numerous changes in alternative splicing triggered by activation of the insulin or wingless signal transduction pathways in *Drosophila *cells in culture. These changes are pathway-specific and affect genes with functions coherent with the distinct roles of each pathway *in vivo*. Thus, carbohydrate, amino acid and intermediary metabolism genes are enriched among the targets of insulin, while components and modulators of signal transduction are enriched among wingless targets, which, interestingly, include also genes important for memory and olfaction. Forty percent of genes showing alternative splicing changes also showed changes in transcription, suggesting a significant overlap and potential coupling between the two processes upon signal cascade activation. Bioinformatic analyses of the target genes identified sequence motifs enriched near regulated 5' splice sites specific for each pathway. Our results argue that signaling pathways can trigger a coherent set of alternative splicing changes that are relevant for cell growth and differentiation.

## Materials and methods

### Cell culture assays, western blotting and RNA isolation

For insulin pathway activation, S2 cells were grown to exponential phase and 30 μg/ml human insulin (Actrapid 100 Ul/ml, Novo Nordisk, Madrid, Spain) was added to the cell medium. Cells were harvested after 5 hours. The wingless pathway was activated by treatment of 1.5 × 10^6 ^S2 cells with 15 μg of *axin *dsRNA for 4 days as described [78]. For western blotting, samples were fractionated by electrophoresis on 8% denaturing polyacrylamide gels, transferred to nitrocellulose membrane (Schleicher and Schuell, Dassel Germany) and probed with anti-phospho-Akt (Ser473) antibody (1:1000; Cell Signaling, Boston, MA, USA) or anti-armadillo antibody (1: 400; N2 7A1, Hybridoma Bank, Iowa City, IA, USA). Antibody detection was carried out using the ECL-detection kit (Amersham Pharmacia Biotech, Uppsala, Sweden).

Total RNA was isolated from cells following the RNeasy Miniprep protocol (QIAGEN, Venlo, Netherlands) including DNase treatment. The integrity of the RNA was controlled using a Bioanalyzer and only RNA preparations with undetectable degradation of ribosomal RNA peaks were utilized for further analyses.

### Microarray experiments

Microarray hybridization, data acquisition and analysis were performed as previously described [51]. In brief, cDNA was generated from 15 μg of total RNA with the incorporation of aminoallyl-dUTP (Sigma, St. Louis, MO, USA). cDNA was then conjugated with Cy3/Cy5 mono-functional dye (Amersham, Uppsala, Sweden) and hybridized to custom 44k Agilent oligonucleotide arrays. After hybridization, arrays were washed, scanned and images analyzed following the manufacturer's recommendations. General gene expression values represent the average of log2 ratios for all the probes of a locus. The net expression of a splice junction was calculated by subtracting the average log2 expression of all junctions of an isoform from the log2 expression ratio of that particular junction. A Z-score was computed for each value [51] and a Z-score cutoff of 2 was used to consider changes as significant. We define a splicing change as a consistent change in the relative signal associated with a splice junction probe between two conditions, whose Z score is statistically distinguishable from the signals from other probes in the array and from the average change of all other probes monitoring other splice junctions and exon probes in the same transcript. The array data have been deposited in the GEO database [GEO:GSE14085].

### Primer design and quantitative RT-PCR

The design of primers for validation was carried out using the public software primer3 [79]. Amplicons were approximately 100 base pairs. To assess the general transcription level, a primer pair was designed on constitutive exons, while a primer pair covering the splice-junction was designed to validate changes in that junction. cDNA was synthesized using a mixture of oligoT and random hexamer primers from 1 μg of total RNA using Supercript II (Invitrogen, Carlsbad, CA, USA) following the manufacturer's protocol. Real-time PCR was performed for 45 cycles using Lightcycler DNA Master SYBRgreen I (Roche Applied Science, Pensberg, Germany) in 384-well plates using Lightcycler 480 (Roche). Efficiencies of primer pairs were experimentally calculated and specificity of primers was controlled using a melting curve. The log2 ratios were calculated as described [80]. Individual PCR amplifications were carried out in triplicates and analyses included at least three biological replicas.

### Motif discovery

We retrieved 50-nucleotide-long intronic regions proximal to the splice sites of every regulated splice junction and the corresponding orthologous sequences in the other 11 *Drosophila *species using sequence alignments generated by MAVID [81]. MEME [61] and PHYLOGIBBS [62] were used to search for putative common motifs. The statistical significance of each motif was assessed against a corresponding collection of motifs found using the same methods for deriving motifs enriched in sets of control regions. These control regions were retrieved separately from constitutive and non-constitutive junctions mimicking the sample size and phylogenetic coverage of the sequences associated with the regulated junctions (for additional information and statistics, see [63]).

### Gene Ontology analysis

We used the latest *Drosophila *GO annotations provided by the Bioconductor project version 2.4 through the annotation package Org.Dm.eg.db version 2.2.6. We used the GOstats package from Bioconductor to calculate the enrichment of GO terms using the conditional hyper-geometric test [82]. The gene universe is composed of all the genes represented on the array. Overrepresentation analyses were performed for genes regulated at the level of transcription, alternative splicing and those genes with splicing, but no transcriptional changes for each signaling pathway. Only categories with a hypergeometric *p*-value lower than 0.05 were considered.

## Abbreviations

dsRNA: double-stranded RNA; GO: Gene Ontology; hnRNP: heterogeneous ribonucleoprotein particle; UTR: untranslated region.

## Authors' contributions

BH carried out all the experiments presented in this paper and, together with JV, designed the overall content and experimental setups of the study and wrote the manuscript. RC carried out the *in silico *analysis of enriched sequence motifs and, together with SB, provided expertise in GO analysis. SB also contributed the assignment of alternative splicing events. MB and DCR provided splicing-sensitive microarray designs, logistic and experimental support for array hybridizations and expertise in data analysis. All authors provided feedback and approved the final the manuscript.

## Additional data files

The following additional data are available with the online version of this paper. Additional data file 1 is a table listing the calculated Z-scores from the microarray experiments, for each biological replicate, for genes that are transcriptionally regulated by the insulin (top) and wingless (bottom) signaling pathways. Additional data file 2 is a table listing the calculated Z-scores for splice junction probes that are regulated upon induction of the insulin (top) and wingless (bottom) signaling pathways. It also includes the sequence of the probes. Additional data file 3 is a table listing the results from the GO analyses done after removal of genes showing changes in alternative promoters.

## Supplementary Material

Additional data file 1 Calculated Z-scores values from the microarray experiments, for each biological replicate, for genes that are transcriptionally regulated by the insulin (top) and wingless (bottom) signaling pathways. Click here for file

Additional data file 2 Calculated Z-scores for splice junction probes that are regulated upon induction of the insulin (top) and wingless (bottom) signaling pathways. Click here for file

Additional data file 3 The table represents the percentage of enriched GO categories for genes regulated at the level of alternative splicing by (A) insulin and (B) wingless. Click here for file
