# Supplementary material for: Global analysis of alternative splicing regulation by insulin and wingless signaling in Drosophila cells
Source: Genome Biol. 2009 Jan 29;10(1):R11. doi: 10.1186/gb-2009-10-1-r11 (PMC2687788; doi:10.1186/gb-2009-10-1-r11)
Supplement: Additional data file 1 — Calculated Z-scores values from the microarray experiments, for each biological replicate, for genes that are transcriptionally regulated by the insulin (top) and wingless (bottom) signaling pathways. [file gb-2009-10-1-r11-S1.pdf]

cg_list	Insulin_Zscore_ave	Insulin_1_Zscore	Insulin_2_Zscore	Insulin_3_Zscore
CG16833	3,2	3,105451171	3,366304823	3,23091471
CG18076	-2,0	-1,735312649	-2,231090798	-2,169268468
CG4551	-2,3	-2,296373281	-2,408859141	-2,296390729
CG10576	3,0	2,961970565	3,068809719	2,96397203
CG7958	-2,1	-2,145340194	-2,026924185	-2,063913327
CG9281	3,2	3,437529043	3,161275571	2,917890103
CG3978	-2,6	-2,261259772	-2,778362858	-2,643706836
CG1516	2,4	2,693792306	2,104340979	2,264778296
CG5059	-5,8	-6,011860495	-5,788981532	-5,594158921
CG32195	-3,1	-4,436600432	-3,052994249	-1,863727034
CG9520	-3,4	-3,444706082	-3,492509438	-3,132177999
CG18466	7,2	6,73524119	7,505792623	7,234970254
CG4280	-2,4	-2,521584096	-2,306890275	-2,489406187
CG6058	3,4	3,557761977	3,508615586	3,183162707
CG1817	-2,2	-2,06103971	-2,306892441	-2,33002985
CG12919	-2,8	-2,937255358	-2,384754595	-2,979698417
CG31299	2,1	1,941417767	2,192690859	2,052962559
CG30011	-2,1	-2,064833443	-2,242935373	-1,845887103
CG2194	2,1	1,858668492	1,89341224	2,50124459
CG6643	-2,7	-2,755696616	-2,733045064	-2,57587592
CG31543	3,7	3,800052318	3,931523595	3,229280517
CG10971	-2,5	-2,64070539	-2,341492062	-2,58351435
CG6134	-3,4	-3,352297017	-3,531744727	-3,30962027
CG8026	3,1	2,764095785	3,377963535	3,22462488
CG11120	-2,4	-2,663051768	-2,332112722	-2,100633367
CG2086	-2,9	-2,845118587	-3,009472316	-2,879536892
CG9696	2,1	2,337113397	2,120648181	1,88861293
CG10433	-2,8	-2,574398343	-2,884775421	-2,968826928
CG6084	-2,0	-2,194368134	-2,197099781	-1,696485629
CG3647	2,0	2,22223674	1,897124274	1,933730383
CG3168	2,6	2,898122974	2,794102603	2,120958899
CG13213	-2,7	-2,625896644	-2,841569954	-2,666936449
CG31666	-2,4	-2,186044046	-2,444055526	-2,619769923
CG33188	-2,1	-2,058725076	-1,802666153	-2,397431687
CG10021	2,4	2,562000431	2,336826086	2,167013236
CG14080	-2,6	-2,273245349	-2,491931889	-2,898073606
CG1600	-3,2	-3,27832849	-3,190575016	-3,277791586
CG15081	2,1	1,876805301	2,236284506	2,095577919
CG10691	2,2	1,926841258	2,172624209	2,457843433
CG11486	-2,3	-2,233708481	-2,438309561	-2,354589742
CG8118	-2,4	-2,367533467	-2,367424618	-2,326950054
CG32369	3,5	3,97301985	3,630564128	2,968031777
CG4608	5,9	5,804696013	6,216216765	5,598631313
CG18808	-2,0	-1,85058462	-2,128853646	-2,052735111
CG2257	-2,3	-2,201300623	-2,484750509	-2,328894834
CG11661	2,3	2,398129204	2,318590706	2,060239732
CG11652	3,7	3,645365504	3,972374902	3,49945389
CG15154	3,2	3,558645582	3,415108307	2,69637405
CG3992	-3,1	-2,874519551	-3,07871077	-3,456487279
CG32103	2,8	3,035957872	2,797553688	2,617709942
CG3979	3,3	3,479899083	3,227569683	3,200766369
CG4429	2,3	2,486516667	2,605323458	1,936118792

CG3606	2,4	2,025453994	2,781930518	2,252279295
CG9878	2,8	2,810707517	2,95916334	2,682232217
CG15112	-2,7	-2,48876564	-2,492635714	-2,972112231
CG6767	2,8	3,116176436	2,875257645	2,497873716
CG11844	2,8	2,483334876	3,116718792	2,714556532
CG2674	2,1	2,014449374	2,306488257	1,925330724
CG1572	-2,2	-2,070108015	-1,588858865	-2,850132191
CG33199	2,1	2,523679305	1,72209512	2,18441355
CG7421	3,8	3,975127681	3,885875653	3,68693109
CG1743	-3,9	-3,75724377	-3,704088713	-4,308940453
CG9809	3,8	3,968339203	3,865407524	3,570191269
CG5854	3,6	3,252928663	3,716956751	3,714749613
CG14253	-2,5	-2,817351518	-2,615644027	-1,930183293
CG18335	-4,6	-4,324740366	-4,869360706	-4,579185064
CG8095	-2,5	-2,459476471	-2,531504455	-2,613430511
CG3937	-2,8	-2,697737679	-2,740600441	-3,03502957
CG5033	2,9	2,932714201	2,831383331	2,930158846
CG7334	2,0	2,387594905	2,021080518	1,718606219
CG8222	-4,1	-4,183452607	-3,880231824	-4,108686292
CG8355	-2,0	-1,501001324	-2,550349908	-2,024932773
CG1544	2,5	2,487183868	2,362767309	2,737966181
CG3983	2,8	2,698654207	2,611299232	3,094934727
CG6231	3,8	4,006938567	3,489216108	3,806889921
CG4257	-2,0	-1,769418548	-2,230390549	-2,063138863
CG5973	-4,2	-4,160892869	-4,560722576	-3,833512178
CG12128	2,2	2,094485899	2,344570091	2,236902043
CG14792	2,4	1,861130317	2,620110718	2,70508564
CG2092	-2,1	-2,137329195	-2,3612487	-1,904429502
CG2671	-2,4	-2,435886124	-2,246163832	-2,564514385
CG4675	-2,3	-1,923053138	-2,50814961	-2,576238905
CG8327	2,0	1,584921188	2,228756587	2,194616918
CG1799	2,6	2,466344236	2,639665054	2,690573281
CG3001	4,2	4,589941071	4,529634349	3,49832087
CG8098	2,6	2,939772849	2,621550147	2,3582621
CG30035	4,9	5,007274489	4,646242776	4,978034415
CG1106	-2,1	-2,337699877	-2,069648932	-1,882300835
CG33066	2,8	2,845047709	2,668539609	2,914472602
CG11804	-3,7	-3,779516826	-3,778405644	-3,540843167
CG6692	-4,3	-4,649121476	-4,464182954	-3,821820999
CG16944	3,4	3,590118804	3,293716732	3,183385808
CG4620	-4,6	-4,629532492	-4,470590291	-4,668942725
CG12157	2,1	2,507390384	2,172277775	1,726585266
CG1322	-2,2	-1,972683845	-2,482778095	-2,278557385
CG6016	3,1	3,628487631	3,026162847	2,761899514
CG3962	2,6	2,601402244	2,79242601	2,307263436
CG14981	2,2	1,905569491	2,205342103	2,433011641
CG9239	3,3	3,546682339	3,326219405	3,161526916
CG31641	-4,5	-4,53712358	-4,797099253	-4,296779235
CG4822	-2,3	-2,406302251	-2,363863721	-2,08454972
CG8256	3,3	3,518012942	3,181263627	3,348691644
CG15611	-2,6	-2,285623345	-3,111357925	-2,335105315
CG17077	-2,8	-2,39078781	-2,747790758	-3,24057007
CG1746	2,4	2,359951943	2,129989946	2,697882374

CG1771	-3,2	-3,15476271	-3,261561966	-3,194053586
CG15009	5,4	5,412679047	5,530351189	5,225424766
CG6794	-3,3	-3,115339869	-3,198291842	-3,495533417
CG12437	-2,6	-2,388632013	-2,798558216	-2,709762542
CG11347	-2,4	-2,179787732	-2,800610509	-2,331392084
CG18619	-4,6	-4,599057662	-4,652298502	-4,594537488
CG1128	2,1	2,171628234	2,020939299	2,070954848
CG9160	2,1	1,81629599	2,177712646	2,387105073
CG11466	-2,3	-2,345643173	-2,14578281	-2,328079048
CG8368	2,4	1,849798919	2,785308886	2,510667161
CG7950	3,2	3,019254392	2,58710467	4,054803752
CG11660	2,1	2,196502719	2,258579773	1,897252428
CG12008	-3,1	-3,018815208	-3,058896126	-3,361348349
CG17888	2,6	2,768416973	2,58098643	2,562817034
CG10082	-2,2	-2,173612656	-2,361463751	-1,99388155
CG17836	2,5	2,668123633	2,605653453	2,24240631
CG5320	2,5	2,253555652	2,716192694	2,400934908
CG9328	-2,7	-3,252007566	-3,132661147	-1,618608357
CG17734	3,3	2,939639502	3,295286891	3,655795438
CG18135	2,3	2,14208235	2,988984098	1,817616107
CG15547	-2,2	-2,007161815	-2,72641645	-1,860610389
CG9535	-2,3	-2,688259673	-2,057006581	-2,288034761
CG1404	2,0	2,296565481	2,061983763	1,742029246
CG2206	-2,1	-2,262908657	-1,92272406	-2,057220845
CG10645	3,1	2,862432789	3,358472096	3,186682879
CG33464	-2,2	-1,451119698	-3,253269975	-1,901066525
CG30084	-2,3	-1,899495427	-2,594601548	-2,375758572
CG30492	-2,1	-2,004158642	-2,39534953	-1,896134697
CG10922	2,4	2,123235965	2,41134814	2,520817629
CG10120	2,2	2,419971895	2,394116665	1,819449906
CG10365	-2,4	-2,570280971	-2,444214845	-2,33403192
CG3808	3,9	3,653496168	4,51248785	3,651087083
CG8772	5,5	5,526268102	5,477333417	5,444669108
CG31732	-2,3	-2,438929937	-2,186700531	-2,317195613
CG3376	-5,0	-5,255371239	-4,856677849	-4,874663253
CG6647	2,3	2,501758046	2,177108567	2,312669035
CG10334	-2,3	-2,318691791	-2,168051229	-2,41484844
CG16758	2,7	2,444467792	2,711987307	3,04917374
CG8448	-2,2	-2,122132228	-2,416796136	-1,947685097
CG17608	4,3	4,294737641	3,908055752	4,744229572
CG4912	3,9	3,565219354	4,182357262	3,982204691
CG30015	-2,8	-2,446894042	-3,00980579	-2,912875498
CG7777	-2,4	-2,678455703	-2,224902019	-2,208858481
CG1770	-2,8	-2,666272754	-2,686349829	-3,117360113

cg_list	Wingless_Zscore_ave	Wingless_1_Zscore	Wingless_2_Zscore	Wingless_3_Zscore
CG4551	2,3	2,22952478	2,056482669	2,634844049
CG10497	2,8	2,723342699	2,670947025	3,149967717
CG1225	-2,5	-2,531075812	-2,292366995	-2,661755243
CG8398	-2,2	-1,875529215	-2,430644628	-2,251512158
CG9520	-3,0	-3,056375345	-3,182530234	-2,802619281
CG2139	2,1	2,052962955	2,247149847	1,850970331
CG12919	-3,1	-2,549242754	-2,978951404	-3,839708783

CG32626	5,3	4,341979488	5,64220315	5,907631091
CG9155	-3,1	-3,439632262	-2,89602042	-3,108836953
CG3625	3,1	2,40695948	3,376506797	3,394865256
CG9739	3,8	3,944423936	4,457363618	2,991359413
CG2086	-2,1	-2,539058909	-2,476376614	-1,266292605
CG10433	4,9	4,76309151	5,090065709	4,962246691
CG5201	2,9	2,948881824	2,813128136	2,932321128
CG32434	2,2	2,392338918	2,358449274	1,97470567
CG18279	-2,2	-1,672948422	-2,289664605	-2,652597708
CG5547	-2,3	-2,310749455	-2,150226503	-2,423779033
CG4608	4,8	5,706860424	4,499862989	4,047696962
CG32666	3,0	3,019442764	3,316922979	2,560944721
CG10579	4,8	4,849122629	4,508776046	5,109989851
CG11661	2,5	2,759420163	2,28654044	2,553979867
CG10741	3,3	3,079656486	3,698540385	3,148910534
CG16987	4,4	5,344648743	4,492064708	3,390639526
CG13076	21,5	20,10738673	21,56889803	22,69646746
CG7926	-4,9	-4,815440314	-4,366069494	-5,425365157
CG1572	3,2	3,625794893	2,413621669	3,52166242
CG6030	2,2	2,602666222	1,872068122	1,97796022
CG33199	2,5	2,841304036	2,335129773	2,175577182
CG14253	-4,0	-4,503104787	-3,921943651	-3,445667199
CG5393	-2,1	-2,532667462	-2,330486879	-1,409719476
CG6543	2,3	2,670469913	2,198584962	2,085579787
CG17090	2,6	2,389225832	2,71226554	2,578326595
CG33130	2,2	2,198440434	2,189071591	2,127160163
CG3937	-3,5	-3,227086778	-3,77022883	-3,638128449
CG8222	-2,1	-2,266509651	-1,958023476	-2,218930483
CG13384	-2,6	-2,575405609	-2,86164492	-2,335322468
CG7981	-2,4	-2,303600532	-2,172761024	-2,633522585
CG18408	2,3	2,309013218	2,180718843	2,445729858
CG6953	-2,3	-1,346946758	-2,390245511	-3,269430417
CG5992	4,5	4,544941741	4,396542752	4,691742706
CG8468	6,6	6,292655312	5,982623623	7,442445893
CG3395	3,1	3,93219983	2,510086754	2,964052103
CG9350	2,2	2,131742043	2,138674476	2,359745801
CG4212	2,7	2,830143575	2,216652636	3,039213308
CG5825	2,3	2,759540457	2,423287505	1,667512857
CG11804	3,0	2,886321736	3,069829004	2,917067459
CG6692	6,2	5,406557039	6,559680843	6,783626393
CG2747	5,6	5,477049036	5,89411143	5,379320739
CG7340	-2,0	-2,089502934	-1,439797495	-2,480301434
CG7023	2,2	2,403937304	1,816446954	2,476555121
CG17383	6,3	5,50439071	6,558029886	6,721768521
CG7147	-2,0	-1,741625244	-2,603297865	-1,708680369
CG1322	3,4	3,455944283	4,213470997	2,554446065
CG33207	2,3	2,567419641	1,870236549	2,611271979
CG17932	-3,0	-2,565514279	-3,227514983	-3,243239372
CG7123	-3,6	-3,499048291	-3,782836062	-3,503014617
CG33232	7,4	7,453866313	8,596413973	6,152813582
CG4822	3,0	3,567275168	2,735440521	2,684337578
CG8256	5,5	4,62663481	6,033932928	5,732085159
CG15611	-3,8	-3,837295757	-3,983675312	-3,690221223

CG4859	-4,7	-3,053974653	-5,301607766	-5,692813962
CG11988	2,2	2,449257223	2,030513576	2,126399264
CG12002	-2,6	-2,315483875	-2,130395344	-3,334666014
CG32306	3,9	4,162562588	3,510515092	3,907947059
CG7935	-3,6	-5,464580781	-2,909613172	-2,532173319
CG15828	2,8	4,157682624	2,649064077	1,596786159
CG10082	5,0	4,832925096	4,772287954	5,245412055
CG1630	3,0	3,13635646	2,568388173	3,380020335
CG1471	2,7	3,080126381	2,681995573	2,243311791
CG6449	-3,1	-2,877419508	-3,562338082	-2,824799504
CG30084	-2,1	-1,802086673	-2,086977176	-2,298761206
CG10960	6,1	5,346790483	6,25544976	6,79730764
CG32066	-2,3	-2,329547216	-2,080912385	-2,393893896
CG4692	2,2	2,190796402	1,863343954	2,463570037
CG5923	2,1	2,32146712	2,774242251	1,081308508
CG10365	2,3	2,411475257	2,242209527	2,195256399
CG8588	-2,1	-1,773741601	-1,702811107	-2,864791527
CG3376	-2,5	-1,911991971	-1,682589393	-3,778784526
CG16758	2,1	1,41495521	2,164250959	2,841454658
CG11198	2,9	2,285656837	3,803363757	2,546613509
CG17608	-3,9	-3,388382474	-3,864140774	-4,458166541
CG32498	9,2	8,458419288	9,200246636	9,803328302
CG7740	-2,4	-2,489591838	-2,381307653	-2,311110801
CG30015	3,8	3,340010704	4,156950511	3,866605747
CG1803	-6,9	-6,656458387	-6,023801152	-8,035843955
