# Supplementary material for: Global analysis of alternative splicing regulation by insulin and wingless signaling in Drosophila cells
Source: Genome Biol. 2009 Jan 29;10(1):R11. doi: 10.1186/gb-2009-10-1-r11 (PMC2687788; doi:10.1186/gb-2009-10-1-r11)
Supplement: Additional data file 2 — Calculated Z-scores for splice junction probes that are regulated upon induction of the insulin (top) and wingless (bottom) signaling pathways. [file gb-2009-10-1-r11-S2.pdf]

ProbID	Insulin_avg_Zscore	Insulin_1_Z	Insulin_2_Z	Insulin_3_Z	cg_list	probe_table::seq
2503	-3,795213953	-4,41	-4,27	-2,71	CG16833	TCACAAATCGCGAGGCAAAGTGGCATACTATGCATT
2167	-2,67428805	-2,27	-2,64	-3,11	CG3779	GATCCCGATATATATATGATCGCGCCCATCAATTTA
9803	-3,182700166	-3,50	-2,53	-3,52	CG7450	TTTCTATGAAAATCGAGTAAAAGCGCATGCAACAAG
13105	3,047838566	2,33	2,76	4,05	CG3937	TGGACGGACTTGAGCCAACTGACTTGGCGGTAACCA
19591	-3,071113543	-3,81	-2,73	-2,68	CG3725	GATTGGATTGGATAGGTGGCCACTGAACGAAGAGGA
19589	-2,791024802	-3,41	-2,19	-2,78	CG3725	AACAACTTGATTTATAAGGCCACTGAACGAAGAGGA
690	2,571097436	2,84	2,38	2,49	CG3365	ATTCGAGATTCAAACGCAGCGCGGTCTGACGCCGCC
17582	2,834298354	2,52	3,19	2,80	CG18076	GCCGAGAAGCGGATCAAGCAAGCAGTATTCGAGAAG
7742	3,360529791	2,67	3,92	3,50	CG11486	TATCTTTTGAGTTTTCAGATACGGCTGCTATGGATC
6166	-2,375902553	-3,18	-1,85	-2,10	CG9177	CATCAAATATACAATAAGAAACACCAGCAAAAAAAA
6943	-2,533491916	-2,12	-2,14	-3,34	CG1417	ATAAATAATAAGAACGAACAACAACCGAGATTGGAG
12702	-2,540219015	-2,20	-2,20	-3,22	CG3731	GCGCAGCGCAGTACATTGGCTATTGAACGAATTAAT
7640	-3,040893764	-3,14	-2,90	-3,08	CG16758	GGTTCTCAACGAAGACACGTATCCCTATGAGGTCAT
3512	3,962668583	4,69	3,39	3,81	CG10334	AGCTAAATCCCAAAGTAATTTTCTAAACACACACAC
9940	-2,396322188	-1,83	-2,45	-2,92	CG4899	CTGGCCGCTGGAGCAGCGAAGGTGGCCATCATTGAT
11759	-2,984943773	-3,03	-2,84	-3,09	CG18466	AAAAGAAGTCACAATAAGCAACATGGCTCAAATTAT
4877	-3,989334153	-4,13	-4,15	-3,68	CG3168	AGAGAATAGCAACAAGAAACCGCTAAGCGAGGCAGA
17509	-2,8185317	-3,32	-2,64	-2,49	CG4832	CTCGGATTTACAATAAATAGTCGCTGCAAATATCTA
7487	-2,968924539	-2,33	-3,43	-3,14	CG7995	AACAATCGAATACAAAAAGCAGCATGACCGAGGGCA
19194	3,111642673	2,74	3,50	3,10	CG13499	CACAGAAACGAGGTAGGTCGGAGGTGGAGGCGGCGG
7708	-2,423686269	-2,00	-2,92	-2,35	CG2103	ACATATGTCTGCGATAAGACCACGTTGCAGTGCCGC
14212	-2,566472275	-2,24	-2,99	-2,47	CG5854	CTGTTCTGATTTTGGGTGGCTGCGGCTTCATTGGAC
7483	-2,712896373	-3,16	-2,94	-2,04	CG7995	GGGGCAGTGCTAATGTGGGCAGCATGACCGAGGGCA
7644	-4,771924473	-4,16	-5,00	-5,16	CG16758	CGCCCAGGAGAATCCCACCGGAAATGGAAAAATCCA
3509	3,527618602	3,77	3,33	3,48	CG10334	AGCTAAATCCCAAAGTAAATACACACATCACCCCTA
15811	-2,34893186	-2,43	-2,50	-2,12	CG8276	TAGGTGTTTTCTAACGTTGGTACATCTCAACGGAGT
15318	-3,337784673	-3,28	-4,04	-2,69	CG7950	ATATCTGGACGTGAAGATATAATTCACGTTTTTAAT
6948	2,563643434	2,73	2,09	2,88	CG1417	CGAGAAACGTGAAGTTGAATCTTCCGTCTCCAGTGC
3638	3,236900476	3,48	3,03	3,20	CG9326	ACGATACCTCCGATAACGCCGCCTTTCGCAACTCGA
7037	2,776081304	2,37	3,33	2,62	CG1210	AATGTGTGCTGCTAGCTGTTAAAGTATGCGAGAAGC
1137	-2,780215648	-3,22	-3,09	-2,03	CG10021	TCAAAATTGTCCCTTAGTAATCGTATTAACAGAAGT
14087	-2,606481584	-2,93	-2,66	-2,23	CG17894	GAGCTTCAACAAGATAAGGACAAGAACAATGAGAAT
16498	3,767050837	3,45	3,94	3,91	CG11804	AAGTAGCGGACCGTGAAGCTCCCTTAAACGCTTAAA
8446	-2,454349543	-2,19	-2,45	-2,72	CG8588	GGACGCGAGGTGGGAGCGATCACCCAACTGACTGTG
18801	-2,450058138	-2,69	-2,21		CG8201	AAGAAAAAAACAACAGAAAATTTAACAAGATGGTCT
13310	-2,670706202	-3,55	-2,16	-2,30	CG18617	TAATTCTCGTCGGCGGAGGGAACGTAAGCAGTCGCC
17075	-3,339648543	-2,85	-4,08	-3,09	CG30035	ATTTGTACCTTCTCCCAGGTCTTGGCTGCTCTGAGT
8987	-2,389301993	-2,83	-2,62	-1,72	CG6767	TACGATTGGAAATTTAGGAGTCGTGCGCCCATCTCG
6016	3,25542479	2,59	2,14	5,04	CG8948	AGTCCGTTCTTGATGAAAGAAGGATTACCAATATGC
10045	-2,493151751	-2,24	-2,61	-2,62	CG9674	AAATAATCTACGATCACGTGTGATTGTTCAGGCTGA
10094	-2,785409147	-3,51	-2,52	-2,33	CG11661	ACTCTGGGTTTTTGTTTGGCGAGCAGGACATGGATC
13446	3,123692428	2,66	3,60	3,12	CG31175	CAAGCGGTGGCATTCAGGTGACCCGATTGCGAGAGC
11407	2,988915513	3,07	4,03	1,86	CG10279	CGGCAGCATGCGAGCAAAGGCACCACACGATCGCGA
9145	-5,191961027	-5,70	-5,18	-4,69	CG7958	GAAGCGCAATTCCAAAAAGAAAAATGATGAAATGAA
16895	-2,4421665	-2,48	-2,30	-2,54	CG2368	AATATAAATAGCGAAAAGTATACTGGAGTATACACA
10602	-3,135024026	-3,23	-2,72	-3,45	CG5683	CAGCCACAAAATGGAAAGGCACTAGCAGCAACAACA
17593	3,272021661	2,43	3,63	3,76	CG18076	TCCACGCAGCGCAGAGAGATCTCCGATATTGTTGTG
16589	-3,222170303	-3,33	-3,14	-3,20	CG1516	TATGGTACGAAAAGTCAAATACATTTAAGTCCTAGG
8445	-5,298595167	-4,16	-6,79	-4,94	CG8588	GGACGCGAGGTGGGAGCGAAAGCAAAAGACGGTGAC
14882	2,591733474	3,50	2,88	1,40	CG14253	AAGTGGTACGCTACGAACATATAGTAATATGTCTCA
7740	4,490538245	4,16	4,66	4,65	CG11486	ATTATCAAATGCCAGTGTATACGGCTGCTATGGATC
1188	-2,330284038	-2,19	-2,87	-1,93	CG3714	GGAAACGGATCAAAAAACCGACAATTGCAAAACAAT
16060	4,079590883	3,61	4,77	3,87	CG1600	CAATTCACAAGTAAAAGTTGGAACAACAGATACCTC
5723	3,043356608	3,09	3,08	2,96	CG1771	ATGTTCGGATATCACATTTGTTTTAGTGGGAGCTCC
2091	3,160478506	2,64	3,86	2,98	CG12437	GGTTAAATTATTCCTCAGAGAAGTTCTGAATGCATG
16171	-2,43895671	-2,97	-1,36	-2,98	CG1882	CATAGCCAGCGGCTAGAGGATCCCCGGAACAATAGA
18456	3,701203422	3,73	3,99	3,39	CG33130	CGAAGGCGGACGAGAGAGGCGATTTTGTCGCTGCGG
14959	2,985080559	3,19	3,34	2,43	CG5643	AAGTCCGGATGCTCGAAGTGAAAACCAGCAAGCAGC
3052	2,998508471	2,34	2,90	3,75	CG31732	GAAATACCGAATTTCTTGCTGCACGAGATCACCCGG
1136	-3,004476806	-3,47	-3,17	-2,37	CG10021	GCTAGTGTGTGTGTGTGTAATCGTATTAACAGAAGT
12382	-3,650474057	-5,27	-2,88	-2,81	CG10120	AAATTTCGATGAAAATGGCACGCCGGTTTATCCCAC
13470	-6,316767204	-6,66	-6,03	-6,26	CG6231	CATATATAATAACTACAGACCAAGCCAAAGGAACAA
19102	-2,356950707	-2,15	-2,83	-2,10	CG9696	CAGCCCACAACGCAACAGCCATGAGCGATCTAACTG
9171	-2,38273921	-1,89	-2,40	-2,86	CG7888	ACAGCAACAACAACACCATCATGACCAAGAATGGAC
5447	-2,627428631	-3,03	-1,86	-2,99	CG1743	GTGCATTGAAAAAATCAGTATGTCCGCTAGGATCCT

12780	-5,279569592	-4,25	-6,31		CG4898	AATTACCCAAAAATTAAAAAAAAACACAAGTGCAGC
15158	2,733985346	3,05	3,70	1,46	CG2005	GTGGCCATGCAAAAAGAGTGCCAAAATGGAAATCAT
16280	-3,209571573	-3,36	-3,34	-2,93	CG11546	CGACATCAAAGCACGAGAATCAGTAACAGGATCAAG
6937	-5,139591129	-4,36	-6,33	-4,72	CG32527	GGCAATAATATCATTCAACGTAATATGTTTGAGGAT
5804	2,67894396	2,57	2,44	3,03	CG11111	AAATCCGCGGCGCAGAAGGTACCTGGAAAGAAAGAC
14530	-2,633999259	-3,54	-1,72		CG13636	AGGAACTTATTTTTACATTTAAAGGACGAGAGTGCA
3507	-3,03906259	-3,52	-2,79	-2,81	CG10334	GGGTGTTTTTGTTGTCATATACACACATCACCCCTA
14900	4,030980834	3,56	4,26	4,27	CG6134	ACTATCCGGACCTTTCAGACGAGCGATTCCTTTGCA
5829	3,073891493	2,89	2,20	4,13	CG32626	GCGACATGTAAGTCGCCAGTGATCTCGCCGATTCAC
13095	3,378130006	3,71	2,79	3,63	CG3937	CTCTCTAGCAGAGGAAAACTGAAGTCAGAATGCCTA
13473	-5,807987829	-6,07	-4,97	-6,39	CG6231	CTTGCGGCGCATCTCTGGACCAAGCCAAAGGAACAA
14221	-3,431097273	-2,87	-4,23	-3,19	CG5320	GCATGTATCACCTGAAGAATCAACCCTAAGGAGTAC
10603	-4,115304625	-4,11	-3,98	-4,26	CG5683	AAAGCCCGCCCAAAAAAGGCACTAGCAGCAACAACA
14905	2,673017648	3,07	2,78	2,17	CG6134	CCAGATCAGTACTTCAAAAGGACGGACACGGAGGTG
7738	4,002530412	3,12	4,33	4,56	CG11486	ACAATTTCTTCCCGGCAGATAATGTGCTCGACTTAT
12712	2,922603068	2,78	2,45	3,54	CG6904	GTCGACAATTGTTCAACTATGAATCGTCGCTTTTCG
13220	-2,900162065	-2,24	-3,24	-3,21	CG31243	CCGAATAATAAACAAACTAAGCATCAAAGTGAGGAT
10488	-2,622396458	-2,68	-2,53	-2,66	CG32425	GTGGAAACAAGGCAGCAGTTAATTGCAACTCGAAGG
3791	-2,902628114	-2,53	-3,65	-2,53	CG2201	CAGCATTTCAATAGCAATGCCACCCTTGAGGAAATT
14411	3,474927353	2,82	3,66	3,95	CG6643	TAAGTAAACCGTCCTAAGCGATAACAGTCCATCGGT
17622	3,528885034	4,40	4,37	1,82	CG6692	TCGCAGAGGGCACAATTGATTCCAGAACAAATCACA
16775	2,616832148	2,03	2,64	3,18	CG18408	TACCGCCTTCCGTCCAGGCCCGTCCGTCGTACAAGA
7616	2,698857208	3,10	2,84	2,15	CG32306	ATTTTCCCCGATATCGCAAAACCATTGCGGCCTCAA
5349	-4,976662377	-4,62	-5,75	-4,56	CG16944	GAAACACAAATACAAATTTGAATCGTACATCCACTA
18568	-2,675007718	-3,37	-2,56	-2,10	CG5119	ATCGAGCGTGTTTGAGAATGGTCTAGGAACTAGTTG
2490	-2,532351346	-2,99	-1,32	-3,29	CG6647	TTGTGGTCATGTGAGGAGATGGCTCCTCCATCATAC
10487	3,187999461	2,84	3,81	2,91	CG32425	TCTCCTGCTTAAAAGCCTGCACCGCCAGCAACGTCG
13090	-3,023117988	-3,36	-3,39	-2,32	CG3962	CCAGGGAGAGCAATGTAAAGGCTGAGATGAGCAACA
14216	-2,455476117	-2,33	-2,28	-2,75	CG5320	AAAGAATCATTAATGACGAATCCGTTCAAGAGTCCC
13604	3,653438036	4,29	3,08	3,59	CG4257	AAAAAGGCAATCTGAAAGTTTCCTCCTTCTAACCAT
14901	3,553918469	3,57	4,05	3,04	CG6134	CATGCCCATACCCACCCAAAACTTCTGCACGAATGT
16789	-2,73167048	-2,30	-3,08	-2,82	CG18408	TGAAGGTAACTTGTCCAGAGCTTTGGCCAAGGAATC
7641	-2,433503597	-3,41	-1,38	-2,51	CG16758	AACTCCAGAAAGTCTGCTGTATCCCTATGAGGTCAT
13610	-2,693055443	-2,70	-3,03	-2,35	CG4241	GTGCTAACAAAAACAAAGGTCCCCCAGTCCGAGTCT
13748	-2,449587037	-2,33	-2,57		CG17299	GATATAAAGATTTATTAGTTTTCGGCGAGGCCGACA
9424	-2,407906253	-2,94	-2,16	-2,13	CG32103	CGCGGGGAAATTCGATACACGACTGTTTTTCCATTT
8035	-4,679520129	-4,62	-4,33	-5,09	CG15009	CCCTGATTAATGGAGGCGAAAATGAATTTACATGTG
9292	-3,792635473	-3,06	-5,01	-3,31	CG11658	AGCCACAGCAATTTCTCGTTAAATCAAACGCGTTTC
5143	-4,032638321	-3,92	-3,29	-4,88	CG12737	AAATATATCGAGCATAAGTCCCAGCTTGACGGGCAA
17569	2,57695391	2,69	2,46	2,58	CG18076	AGAACGATCCTTGTCGCGCCGACGAACACTTGGCCG
5325	3,864612934	3,30	4,79	3,51	CG33175	GTGATGGATGCCATAGCGCTGCTCAGCTCGCTGGCC
2869	-2,346999154	-1,93	-2,48	-2,63	CG16885	GAGCGAACCAACCAACAACAACCGCCAACGTACGAC
18566	-2,42615383	-2,55	-2,09	-2,64	CG5119	CATCGAGAACGAAAAGAAGTAGACCTGATCACCAAA
10494	3,010928025	3,04	2,96	3,03	CG5059	CCGGCTAACGAATCGAACAATCTTGGATCGAACTCA
15545	5,986221963	6,27	6,37	5,32	CG11525	AAACGCAGAGGCATCCGGCTCGTAAGTTTTATTTGA
11080	2,853559026	2,79	2,87	2,91	CG9765	CAAGCCATGAGTGGCGAGCTGAGTCCGTTGGCGGTG
17917	2,682192596	2,52	3,34	2,20	CG30084	ACGGCTGTCTGTCAGAAGTGCCCACAGGAGCTCAAA
8577	-2,462798336	-2,68	-2,25	-2,46	CG17888	TTCAGCCAGCCAGCTCAGGCAGGAAATCTCCGTTTT
647	2,552030255	1,94	2,34	3,38	CG4280	CGCATATAAACGCTTCTGGCACCAAAGGTAAAGGGA
16793	-2,557762717	-2,50	-2,12	-3,05	CG18408	ACACCGTCGACGGATCAGACGTGAACATCCACTTCA
532	-2,969276067	-2,95	-3,27	-2,69	CG2674	CCTTTGTTCAAAACCACACTCCACCAACAATTTAGC
18850	-2,403334858	-2,47	-2,11	-2,63	CG9854	CTACACAGGAAGAAAAGGCATTTTATTAAGCGCAGT
12485	-2,378522543	-2,84	-1,68	-2,62	CG8863	TTCTACAAAAAAAAATGGTAAACAGCAAACAAAATG
7737	3,91967329	2,95	2,99	5,82	CG11486	CTCATTAAAGTAATTACGTCGACGCGCCTCAATTGT
11687	-2,379896395	-1,56	-2,26	-3,32	CG8036	TAGAGGTGACCAGCTCAACCATCCCACATCATGTGC
537	-3,10278466	-3,17	-3,08	-3,06	CG2674	AAGAATACACCCCAAATGACAAAATGTGCGACCAAA
6430	-3,177480716	-2,66	-3,44	-3,43	CG5172	ATGGAGGTGGCAATCAAGGTGGCCACGGAGGACAAG
15542	-3,603564985	-3,55	-3,47	-3,79	CG11525	CAAAATGGCTGTAAAAACGTACTAGGCCAGCCCTCA
17344	-6,052627427	-6,49	-5,55	-6,13	CG8772	AGAGGATAATGCCAAGCTCAGTCAGAGACGCCACTA
2634	-3,759659553	-3,57	-5,08	-2,62	CG6756	GTAAAGTTTTAAAAAAGTTTCATCACACATAACACA
16907	3,264944413	3,88	3,39	2,52	CG2368	CTGGCTCTGGCGAGAAAGATGCATTAAAGAACCAAA
16989	3,778521638	4,03	3,62	3,68	CG13213	ATTGTGCGCCTTTAAAATAAGAGCGAGTAGAGTGAT
10091	-2,655696096	-1,89	-2,71	-3,37	CG11661	TTCCGCAGCAACAGCTACCCCTTGAACCAGATCTTC
14427	2,958310599	2,92	2,93	3,02	CG13624	TCGTTTTTATACATTCAAGTTGGCATTTAAATAGTG
19932	-2,326499812		-2,70	-1,95	CG9380	CATTGTTGACTATATAGCTTATGTTATACAGCAGCT
11294	3,13772617	3,34	3,54	2,53	CG2922	ATATCCTTGGGTGAAAGCCAATTTATTGTATGAGTC

17431	2,616825842	2,52	2,91	2,42	CG17054	AAGCTGATGATCGGCAATGCTTTCCGGCGCAACATG
1953	3,147290607	2,95	3,13	3,36	CG8222	CGATCAATGAACAACAATTGCCTTTGCAGCAGTTCT
7633	-2,420644152	-3,47	-2,05	-1,74	CG1146	GACACCACTTGAAGAAAGAGTGATGTGATGTCATAA
17807	-2,866357282	-2,29	-2,65	-3,66	CG8095	GAGCCTGCGACCCAATGGCATCATTGTGGGAGCCCC
4375	2,547567704	3,51	2,26	1,87	CG7981	TGAGGAAGCGCAGCTTCGCGGCTACCCCGCCACCCA
3840	3,374365514	4,14		2,60	CG2238	CGACTGTGAAGACTTAGTGTCAACTTCACCGTCGAC
1949	2,853186433	2,92	3,40	2,24	CG8222	GAGATGACCAGCATAGAAAAGACGGCAAACCGCTGC
5326	4,344231981	4,11	4,86	4,06	CG33175	GGCATTGGCGTTCCCCTGGCGGCAAAGGAGCACGTA
10339	-2,561657213	-3,06	-2,65	-1,97	CG8533	CCGGTTATATATTCCAAGATCAACCTAACATCTTGG
12381	-3,352454149	-3,29	-3,28	-3,49	CG10120	CAGAAAAACTTTTGGACCCACGCCGGTTTATCCCAC
13471	-4,577166928	-4,47	-4,81	-4,46	CG6231	TATCTGGCCTTTAAAAGTACCAAGCCAAAGGAACAA
13509	-4,42167155	-4,37	-4,81	-4,09	CG4608	TTCGCTCTACTACGATTTCCTGTGTCTGCAGCTAAT
5033	-5,198272891	-6,16	-5,18	-4,26	CG2252	CATATGAAAAAGCTAAAATTGCAACTTTTTAACAAT
13410	-2,575963902	-2,44	-2,50	-2,78	CG5558	GGAGCCAAAGAAAGAACACTTGGCGGAGGCCTTGGA
1364	2,705674283	2,72	2,39	3,01	CG14029	AGTCATAAATACGGCTAATTCCCCGCATCCAAGCTC
8574	-2,962225374	-2,88	-3,06	-2,94	CG17888	TTCAGCCAGCCAGCTCAGGCAAATCCACATCGAATA
2502	-2,422502768	-2,42	-2,60	-2,25	CG16833	TTTGCTATTAGTTATATTTTTTTGCCTCCATTTTAC
15701	-3,979083643	-2,37	-3,74	-5,83	CG30438	ATATCAATAAAAAAAAAACAACATGCTTAAAAAAAT
5346	-3,33794831	-3,52	-3,54	-2,95	CG16944	ATCCATAACATCACAAAGCAAAATGGGCAAGGATTT
15258	-2,584601538	-2,39	-2,87	-2,49	CG7816	TGTTACAGCTGAATAACGCCGAGTCATCATCGATCA
17341	-4,420463106	-4,42	-4,86	-3,97	CG8772	CCACTATTCGATGCGGCCCCATCGCGAGCAGGAGCA
10722	-2,545502643	-3,42	-2,03	-2,19	CG7145	GCCTGAAACTCACACAAAATAGCAAAATTCTAGGTG
12970	2,619935724	2,80	2,73	2,33	CG6963	TTCCACGGAGTCCACCGGTGGCGGTGGCCCACAAAC
16880	2,537551015	2,29	2,65	2,67	CG12052	AGCAACGGGATCCCCAAGATTTCGTACTAACCTGGT
10183	-3,424295293	-3,17	-3,85	-3,25	CG3979	TGACTAATCATCACACGGATCTACACGCCACCGCCA
3107	3,212470787	3,49	2,70	3,44	CG12448	TGCATTAAGGGAATCTGATCTGCTAAGTAAGATCGA
7484	-2,614821752	-2,89	-2,96	-2,00	CG7995	GGGGCAGTGCTAATGTGGCATGACCGAGGGCAAGAA
13472	-5,124714494	-5,49	-5,01	-4,87	CG6231	AGAGTTTCTCACAGGCGAACCAAGCCAAAGGAACAA
5803	2,791471964	2,69	3,18	2,50	CG11111	AAATCCGCGGCGCAGAAGGCTGCATGAACAAGCAAC
7481	-3,414504373	-4,35	-2,15	-3,74	CG2086	CTGCAAAAGAAGAGAACTATATAACGTAGACGTTGT
204	-2,80733677	-2,52	-2,46	-3,44	CG1507	ATGGCATCTCAGGATCAAAATATAATGTTGCAAATA
2106	3,341987814	2,98	3,90	3,14	CG9520	AAGGAAGAGCAAACGAAGAAAATGTAAGGAGAAGCT
2814	-3,389205207	-4,69	-2,20	-3,28	CG9239	TGCAGGTCCTTTGCTCAGACGAAGTGTTAATCGACA
1510	-2,361338628	-2,42	-2,38	-2,28	CG31641	GAAAAAGTTCAAAAACTCGAACGTCGCGCTGAGCTC
17806	2,559031267	2,88	2,44	2,36	CG8095	TGTCATCCGCCAGACCAGCATCATTGTGGGAGCCCC
6981	-3,984908466	-3,99	-3,05	-4,91	CG14619	CTAAACGACAGCAGCAAGACGTTGGCGAGAAGTGGC
11685	-3,568917728	-3,28	-2,32	-5,10	CG8036	AAAGCTAATAAGGATAAGCGCCGACGATAGACGATA
5293	-2,352437052	-2,48	-2,28	-2,30	CG32684	AAAGGAGAGCGAAGAGAAAGAAACTGAGAAAACATA
12851	-2,742265933	-2,93	-2,69	-2,61	CG14869	ATGAAGCGTATCAGTGAGTTTGATACGACCACGCAG
13967	-3,629333517	-4,57		-2,69	CG7059	AATGCGCGAAATTCAGGCCTCCCAATTTATGACGAA
13091	-3,433743732	-3,43	-4,48	-2,39	CG3962	AAATAAATAAATACAGATAGGCTGAGATGAGCAACA
10935	2,730819249	3,04	2,52	2,64	CG1106	ACAGCCGTTTCAAATAGACCTGCTTGTGTTTTTGGC
9000	-3,234480971	-2,91	-3,24	-3,56	CG18177	CGAAATGTGAACAAGGAGGACAAGGGCATCCTGCCG
3008	-2,849398639	-2,21	-2,95	-3,40	CG4170	GAAACACTGAAAATTAAGAGCAAAAAGGCAATTAAA
7642	-2,546223694	-2,35	-2,01	-3,28	CG16758	CACTCCACAGAGCCTGCTGTATCCCTATGAGGTCAT
12216	-4,481058199	-4,09	-4,84	-4,52	CG17342	AGGCAGCAAACATGTTAGACGGACAGAATGAGCTGA
1955	2,966861103	2,35		3,58	CG8222	CAGAAACACCCAAATAAATGCAACGCTAAGTGAGCA
8072	4,05059997	4,12	3,16	4,87	CG11347	ACATTGTGGAGAAGGAAGACGAGGAGGACGACTCCC
4879	-4,374000131	-5,24	-3,72	-4,16	CG3168	GCGGAAAACAGCCACAAAACCGCTAAGCGAGGCAGA
5368	-2,609194405	-1,61	-3,16	-3,06	CG1637	ACCCAGTATATACATCGTGTGACGCTGTCCCACCTG
8189	-3,142470765	-2,83	-3,05	-3,55	CG10645	AAAATCACCGAGAGCAAGTTCTTCAAATGGGTTGAC
10961	-3,11168404	-3,61	-2,61		CG32490	AGCTCGACGATTTTACAAAACTGAAAAATCAAATAG
20057	-2,650337348	-3,17	-2,37	-2,41	CG11961	GAGGAGAAAAGATCGAAGGCTCAGCCCAATGGCTGT
10496	-4,536445185	-4,49	-4,32	-4,80	CG5059	GCGAAGATTTGCTGGGCGAATCTTGGATCGAACTCA
10505	-2,934094931	-3,43	-3,47	-1,90	CG4365	TTGGAGAAAAAAACAACTTGCAAAAACTACGAAAAG
3396	2,610177086	3,38	2,50	1,94	CG10473	TTTTCTTATCAAGAAAAGATCTTTCCAGCGTCGCAG
2926	2,923788355	2,86	3,14	2,77	CG4551	AATTGGTCTGTCATAAAGAAATCTTTATGTTTTGCG
3510	2,940877937	3,67	2,66	2,50	CG10334	CGCACACACACACGCATGTTTTCTAAACACACACAC
2082	-2,54615235	-1,93	-2,93	-2,78	CG9310	CTGGCCATTGAGCTTTAATTGCCGGTCATTACTTCA
16159	-2,608931093	-2,66	-2,40	-2,77	CG1553	GTAAAAAAAAAACTCGTGAACCTTCCAGAAATAGAA
4771	-2,330733036	-1,43	-3,34	-2,22	CG3187	GCATCTCGACGGAATCGGATTGACTTCCAAAAGCTT
6334	-2,579082471	-2,74	-2,34	-2,66	CG4429	TGAATAGAGGATACAATGATCGGCCGGCAAATCGTG
11295	3,031413127	2,59	3,57	2,94	CG2922	GCTCATAAGAAAAGAGAGCAATTTATTGTATGAGTC
10184	-2,653012678	-2,38	-2,91	-2,67	CG3979	ACCGAAACTACAAAAATGATCTACACGCCACCGCCA
14162	-2,842067027	-2,65	-3,21	-2,67	CG10365	GTACAAAACCACCTACAAAATCTCAATTGCACTTGG
13096	2,639058007	3,11	2,32	2,48	CG3937	CAATTGTAACACATCAAGCTGAAGTCAGAATGCCTA

7890	2,795182781	2,46	3,01	2,92	CG12008	TGCGAACGAGTTTGCAAAGTTGGGGAAATACACGCT
926	-2,44361289	-2,59	-3,23	-1,51	CG16987	AATGGATTATTAATTTGGACGAAACTTAACGAAAAG
18960	-2,718917707	-2,19	-3,25		CG3216	CATCTGTCTTCTGCAAAAGCAGATGAAGCTCAGCAA
3010	-4,129208439	-4,24	-4,37	-3,77	CG4170	AAAAAGTATCCATAGATGAGCAAAAAGGCAATTAAA
16448	-2,687229368	-2,68	-2,71	-2,67	CG8026	GGGCTCATGGCAGCTTGAGTTTGAGGGCTACAGAGG
1739	4,36512876	3,86	5,12	4,11	CG5973	TTTGTGTGTATTTTTGCGCTTGAACCGGTGAACAAC
9351	4,268197793		4,56	3,98	CG6928	AAACATTTGTTTAAATACAAATAGAAGCCATGAGCC
18221	-2,368499851	-1,85	-2,49	-2,77	CG8098	TGGAGCAACAGCCGCAGAGATGCTTTGCCACCCGGT
16449	-3,081924243	-2,89	-3,73	-2,62	CG8026	ACGCCCAACATCTGCATGCCTGCGTCTTTTCATTTG
12486	-2,486880293	-2,13	-2,99	-2,35	CG8863	AAACTTCAAAAATTTTCGTAAACAGCAAACAAAATG
7734	2,57196327	2,22	3,29	2,20	CG11486	CAAAGTGAACTATGACTGTCGACGCGCCTCAATTGT
8579	-4,551029173	-4,99	-4,11		CG17888	TTCTGAGAATAAGAAAAGCACAATATTTTTTTCAGA
9622	-2,404621855	-3,05	-1,05	-3,11	CG17364	GTGGATGAAAACCAGGAGTATCCTCCCCGGCTGGAA
19723	-3,916302818	-5,18	-3,51	-3,06	CG3318	ATCGTCGTCAAGGATCGTCCAGAGAACAAGAGATCC
11134	-2,708200655	-3,19	-2,15	-2,79	CG31543	GGAACTTTTGGACAAGCGTGAGCGCCGCTATGAGGA
19222	-2,537899103	-2,45	-2,86	-2,30	CG13503	ACAAATCAGCGGGAAACGATGGCTATTCCGCCACCC
17941	-2,639872207	-2,55	-2,60	-2,76	CG8256	TAGCATCCAGAGCACTAGCCCAATATAATACTCGAT
10632	2,896323096	2,46	3,05	3,17	CG7611	TTAAGAAATGCTTTTACGGAATATAATGTGCTATAC
8071	-6,97595766	-6,07	-6,15	-8,71	CG11347	GTTTTGCGCATCCATAAGCTTCTACGCTACGAAGAA
20044	2,54706282	2,29	2,67	2,68	CG11949	AAGTCCTCAGGCAGACGTGCTGATGCTACACCCACC
5322	4,713256613	5,43	4,42	4,29	CG33175	CCAGCGAATGCATCATGGAACAAGTGAACCCATTCC
5350	-2,80375488	-3,13	-1,69	-3,59	CG16944	AGAAGTTATCAACTGAAGTGAATCGTACATCCACTA
12170	-2,519199561	-1,69	-2,96	-2,90	CG31305	GACAACGAGCTGATCACCCTAATTCCCCACCTAACA
11583	-3,024189132	-2,15	-2,98	-3,94	CG32466	ACGCGGTTACCGGACAAGGTGGCCTGGGTCTGGGCG
8989	-3,126236548	-3,61	-2,95	-2,82	CG6767	CAAGATAAAAAAGACAAGTTGGCAGGCAGTGAGGAT
7482	-4,468792572	-4,54	-4,45	-4,41	CG2086	ACCGTGCTTCTTGCCAAGTTGAATCCGGTTTAATCT

ProbID	Wingless_avg_Zscore	Wingless_1	Wingless_2	Wingless_3	cg_list	probe_table::seq
13603	3,001204712	2,88	2,49	3,64	CG4257	GGTGTTTGTGTACCAAAGTTTCCTCCTTCTAACCAT
13105	2,257054194	2,70	2,33	1,74	CG3937	TGGACGGACTTGAGCCAACTGACTTGGCGGTAACCA
17665	-3,542617306	-2,74	-3,93	-3,97	CG8468	GAACAAGAACAGAATAAAACCACATCAAATTCTTTG
4868	-2,221214652	-3,17	-1,70	-1,80	CG4523	AAATTGGCTGCCAGGCAACCCACACAGATATCGAAA
14671	-6,269928408	-5,25	-6,90	-6,66	CG17383	TAACAATTCATGGGTCATATCTCGAATTCGCAATAT
12702	-2,268328007	-3,16	-1,81	-1,84	CG3731	GCGCAGCGCAGTACATTGGCTATTGAACGAATTAAT
13098	2,391799477	2,94	2,89	1,35	CG3937	CTAGTCGATTGCCCAAGTTTGGCTGTCAGAGAAAGC
11643	-4,80426179	-5,15	-5,45	-3,82	CG2747	TGTGCACGAAGTGAACCGATCGTTTATAAACAGCGA
18369	2,452900807	2,55	2,56	2,25	CG15611	TCTGCTGGACAGCGATAGCCACGAGGATGAGGACAC
5315	5,705388651	5,40	5,40	6,32	CG33175	GGCAGCGGATCCCTGGACTGGCGCTCCAGCTGCCTT
12172	2,794898354	2,19	3,20	2,99	CG31305	CCCATCATTCATCTTTCGGCGTCGGTCTGGTGACGC
16498	-2,283236873	-2,15	-1,57	-3,12	CG11804	AAGTAGCGGACCGTGAAGCTCCCTTAAACGCTTAAA
8446	-3,320188427	-3,66	-2,49	-3,81	CG8588	GGACGCGAGGTGGGAGCGATCACCCAACTGACTGTG
15148	-3,298119373	-4,06	-3,06	-2,77	CG11956	ACTGAGGCTCTGGCAACGATTATACTTTCTCTATGC
7614	-3,044670612	-3,85	-2,12	-3,16	CG32306	AATTCCTGGTCTGTGCAGGTGAGTCACGACCAGTTT
8445	-5,765525642	-5,86	-5,13	-6,31	CG8588	GGACGCGAGGTGGGAGCGAAAGCAAAAGACGGTGAC
14882	4,578310197	6,02	2,96	4,76	CG14253	AAGTGGTACGCTACGAACATATAGTAATATGTCTCA
7902	-3,172873749	-3,92	-3,12	-2,48	CG10579	TTTCAAACTGCAGATATTCCGGAGCCGCGAAGTGCG
1536	-3,09135574	-2,87	-3,34	-3,07	CG11050	GGAACTTCTTGCACCCAGACGCCACTTGCACATCAC
12629	-3,963441275	-4,21	-4,60	-3,08	CG8651	GCAACAACAACAACAACTAAGCGCGTCTTGACCGAA
608	-3,926109294	-3,09	-4,14	-4,55	CG3625	CTAACGTTCCTGGATGCGATAATTCAGGCACTGTAC
7901	-3,517977317	-3,83	-3,14	-3,59	CG10579	CAACAAATGCTAAAGATGGCGTCACAATGCGCGAGA
14669	-4,770487098	-3,82	-4,93	-5,56	CG17383	GCTTGTGTTTGTTTGTCGATCTCGAATTCGCAATAT
19110	-3,08026105	-2,83	-2,48	-3,93	CG10497	AAATACGGAGAGGAAAAGCCGCGCGCGCATACGATA
5829	-4,224020762	-3,79	-4,20	-4,68	CG32626	GCGACATGTAAGTCGCCAGTGATCTCGCCGATTCAC
9880	3,357943863	3,24	4,28	2,55	CG32156	CGCCACTGCCTCCTGCGGATTTGTGAGCATAGCTCG
14221	-3,344400782	-4,04	-3,30	-2,70	CG5320	GCATGTATCACCTGAAGAATCAACCCTAAGGAGTAC
12823	-2,59576777	-1,88	-2,39	-3,52	CG31302	GAACCATATCAAAGAGTGAACTCGAAGAAGAACTGA
14453	2,237544108	2,73	1,75	2,24	CG5794	AGAATCTCCTGGAGCTCTATGAGGTGCGCGTGGCCA
7900	-2,954863186	-2,65	-3,08	-3,13	CG10579	GCCAGTGAAAACGTGCAGCTGACATACGCATACAAA
13604	4,333784614	4,69	3,64	4,67	CG4257	AAAAAGGCAATCTGAAAGTTTCCTCCTTCTAACCAT
10880	2,25169292	2,43	2,19	2,14	CG6143	AAATCGCCGTTGCAACACCTTGTTTAAATAATTTCC
13610	2,289498998	3,02	1,17	2,68	CG4241	GTGCTAACAAAAACAAAGGTCCCCCAGTCCGAGTCT
7245	-2,088919833	-2,61	-1,84	-1,82	CG12038	GCGGTGAAGGTAGGTCGCCTGGCCCGGCACAACGAT
7615	-2,850123709	-2,80	-2,38	-3,38	CG32306	ATTATTTTCTGTTTTCGGAAACCATTGCGGCCTCAA
89	2,037958018	2,24	2,55	1,32	CG1674	TCAAGGCGCAATACAAATGCCGTTGCTTCCTCGCCA
16158	-3,329879859	-1,52	-4,30	-4,17	CG1553	AAACTATAAAATAGCCAGAACCTTCCAGAAATAGAA

5742	-3,302626977	-3,51	-2,48	-3,92	CG1630	TCGCCGCCGCGTCTATATAAATACATAGATAGTTGC
14884	3,19192769	3,83	2,41	3,34	CG14253	CCACTGGAAAACACATATCAAGACCCATAAAAATCG
7909	-2,950153858	-2,90	-3,08	-2,87	CG10579	CAAATTGCAGATATGGAGACTGGCGCAGCACGGACA
6222	3,418715777	3,46	2,36	4,44	CG3525	TAGCACCTAATAGCCGTTCTTTTCGTGATATAGTCG
18441	-2,617579328	-2,89	-2,64	-2,32	CG33130	CGCTAATGAATCTGTGCGGTCCTAAACTCTATAAAC
16939	-2,450967934	-2,32	-3,32	-1,70	CG30015	CACAAGTAATTCCGAAAGACCACCAACAAAAGAGAT
18445	-2,055826313	-2,50	-1,61	-2,06	CG33130	ACTCCCTCACTGTAAAAGAGTCACCCGATGATTATC
9879	3,272322824	3,65	3,43	2,74	CG32156	CTCACCGCCCTGCACCAGGCGTGCATAGACGACAAT
10203	9,575485759	9,78	8,44	10,51	CG9739	AACGGAATGGAAAAAATGTTAGACCACGCCGTTTGT
5826	-4,52218419	-3,76	-4,25	-5,56	CG32626	GCCCGATGATTGCAAATGGCAGGCTACAGGACCTAG
7633	-3,467488248	-2,63	-4,88	-2,89	CG1146	GACACCACTTGAAGAAAGAGTGATGTGATGTCATAA
3659	4,261114611	4,83	4,78	3,17	CG9339	GCTTCCAGTTCAGTCCAGGCAAGGAACCGGCACTGC
14674	-5,921695427	-4,88	-6,29	-6,60	CG17383	TAAATTGTATTTCTCGAGGAATGGAGAACCTGTCGC
11642	9,912087559	9,88	9,56	10,30	CG2747	TATCTCCGAGGCGCGCAGATCGTTTATAAACAGCGA
6390	-2,260509237	-2,85	-2,02	-1,91	CG4829	CAGTTATATATAGCTGCGCTCCATCACAATCCCAGC
2183	2,039306272	1,53	2,85	1,73	CG33298	AGCCTCATCAATCTATAGAATCACCGATTATGGGTC
9876	2,567774584	2,45	2,75	2,50	CG32156	GGGAATTTCGCGGGAAAGCAAGCAGGAAAAGAAGAA
14034	-2,480123233	-2,86	-2,02	-2,56	CG7023	ATCAAGGCGGAAGCACAAGAAACGGAACGGGAACCA
13967	3,939545993	2,62	3,34	5,85	CG7059	AATGCGCGAAATTCAGGCCTCCCAATTTATGACGAA
13601	2,692863007	2,83	1,62	3,63	CG4257	GATCGAAGACAGGATAATGTCCGAACAAATAACGCC
17863	2,119487357	1,96	3,06	1,34	CG16801	GACTCCTGCCGCCGCCCTCACTGGGCAGTTTGCCGA
2926	-2,584894262	-2,40	-2,42	-2,93	CG4551	AATTGGTCTGTCATAAAGAAATCTTTATGTTTTGCG
5741	-3,51219625	-3,94	-2,31	-4,29	CG1630	TGGCTCGGGGTCTCGTCTAAATACATAGATAGTTGC
14486	-3,079061231	-3,48	-3,37	-2,38	CG10693	GCAAATTCGATGACTTAGGTGATATCACTCGTGACA
926	-4,634453095	-5,66	-4,31	-3,93	CG16987	AATGGATTATTAATTTGGACGAAACTTAACGAAAAG
6618	-3,081038054	-3,04	-4,19	-2,01	CG6103	GAAGTGGCTACCAAGGATGTGCAGTCCGTGATACAG
9016	-2,485283064	-2,03	-3,17	-2,26	CG8177	GACGAAACACAGAAAGTGCGCCGCGTAAACTAAAAC
11090	-2,696353598	-2,94	-2,88	-2,26	CG31536	GGAAGAGGAGTACACCAGTGGTCAATCCCCGGCGGA
7905	-2,976313212	-3,70	-2,64	-2,59	CG10579	TTTCCGAAAAAAAAGAGTATAATATTTGACCTCGTT
16496	2,434679007	2,17	2,91	2,22	CG11804	CTCCTTCAGCTTGTAACGCTCCCTTAAACGCTTAAA
19154	-3,301749962	-3,64	-3,77	-2,49	CG10433	TGGAGTGCTGCTACGAACTCCGATCAAACCCAACAC
11133	-2,052280448	-2,42	-2,16	-1,58	CG31543	TATAATATAACCGCCGGTTGAGCGCCGCTATGAGGA
10204	-6,322840872	-6,93	-7,12	-4,91	CG9739	AAGCAGTGACAATCAATCCTGGGCGGAATCGAATGA
15145	-2,812630138	-3,05	-2,86	-2,52	CG11956	TAGGCAGCAAAAAGCGCGTTTTGGTAATGAAGTGGT
11134	-2,363468783	-2,93	-2,66	-1,50	CG31543	GGAACTTTTGGACAAGCGTGAGCGCCGCTATGAGGA
19222	-2,94225084	-3,42	-3,04	-2,37	CG13503	ACAAATCAGCGGGAAACGATGGCTATTCCGCCACCC
9877	2,570953852	2,17	2,70	2,84	CG32156	GGGAATTTCGCGGGAAAGGAAAAGAAGAAGCAGCAG
14670	8,6994043	7,78	11,41	6,92	CG17383	GGCGCAGCCTCCCAAAAGATCTCGAATTCGCAATAT
11583	-3,048115377	-4,01	-3,49	-1,65	CG32466	ACGCGGTTACCGGACAAGGTGGCCTGGGTCTGGGCG
16388	2,167164634	1,85	2,05	2,60	CG8224	CATCATCTTCAGCTACAGTTGCATGGTTGTCAAGTA
