# Supplementary material for: Global analysis of alternative splicing regulation by insulin and wingless signaling in Drosophila cells
Source: Genome Biol. 2009 Jan 29;10(1):R11. doi: 10.1186/gb-2009-10-1-r11 (PMC2687788; doi:10.1186/gb-2009-10-1-r11)
Supplement: Additional data file 3 — The table represents the percentage of enriched GO categories for genes regulated at the level of alternative splicing by (A) insulin and (B) wingless. [file gb-2009-10-1-r11-S3.pdf]

A. Insulin

GO term category	Transcription (56)	AS (29)	AS only (19)
Carbohydrate, amino acid and intermediate metabolism	6 (11%)	1 or 2 (4 or 7%)	1 (5%)
Immune response (including antifungal, antibacterial)	12* (21%)	5 (17%)	1 (5%)
Developmental decisions (including tracheal system)	28 (50%)	6 (21%)	4 (21%)
Microtubule organization	1 (2%)	2 (7%)	0
Germline development; Oogenesis	0	7 (24%)	6 (32%)
Behavior, olfaction, memory, learning	1 (2%)	3 (10%)	2 (10%)
RNA metabolism	1 (2%)	0	3 (15%
Signal transduction, lipid metabolism	2 (4%)	2 (7%)	0
Cell death	0	0	0
other	5 (9%)	1 (4%)	2 (10%)

* includes hemopoiesis

B. Wingless

GO term category	Transcription (47)	AS (34)	AS only (34)
Signal transduction, lipid metabolism	11 (23%)	8 (24%)	6 (18%)
Learning, memory, behavior, olfaction	9 (19%)	8 (24%)	8 (24%)
Developmental decisions	15 (32%)	7 (21%)	6 (18%)
Cell death	0	1 (3%)	1 (3%)
Carbohydrate, amino acid and intermediate metabolism	0	0	0
Immune response	3 (6%)	2 (6%)	1(3%)
RNA metabolism	0	0	0
Microtubule organization	2 (4%)	0	0
Germline development; Oogenesis/ Spermatogenesis	3 (6%)	3 (9%)	4 (12%)
other	5 (11%)	4 (12%)	8 (24%)
